# Enhancement of brain atlases with laminar coordinate systems: Flatmaps and barrel column annotations

**DOI:** 10.1162/imag_a_00209

**Published:** 2024-06-28

**Authors:** Sirio Bolaños-Puchet, Aleksandra Teska, Juan B. Hernando, Huanxiang Lu, Armando Romani, Felix Schürmann, Michael W. Reimann

**Affiliations:** Blue Brain Project, École polytechnique fédérale de Lausanne (EPFL), Campus Biotech, Geneva, Switzerland; Laboratory of Sensory Processing, Brain Mind Institute, Faculty of Life Sciences, École polytechnique fédérale de Lausanne (EPFL), Geneva, Switzerland

**Keywords:** flatmap, barrel cortex, brain atlas, coordinate system, open methods

## Abstract

Digital brain atlases define a hierarchy of brain regions and their locations in three-dimensional Cartesian space, providing a standard coordinate system in which diverse datasets can be integrated for visualization and analysis. Although this coordinate system has well-defined anatomical axes, it does not provide the best description of the complex geometries of layered brain regions such as the neocortex. As a better alternative, we propose*laminar coordinate systems*that consider the curvature and laminar structure of the region of interest. These coordinate systems consist of a principal axis aligned to the local vertical direction and measuring depth, and two other axes that describe a flatmap, a two-dimensional representation of the horizontal extents of layers. The main property of flatmaps is that they allow a seamless mapping between 2D and 3D spaces through structured dimensionality reduction where information is aggregated along depth. We introduce a general method to define laminar coordinate systems and flatmaps based on digital brain atlases and according to user specifications. The method is complemented by a set of metrics to characterize the quality of the resulting flatmaps. We applied our method to two rodent atlases. First, to an atlas of rat somatosensory cortex based on Paxinos and Watson’s rat brain atlas, enhancing it with a laminar coordinate system adapted to the geometry of this region. Second, to the Allen Mouse Brain Atlas Common Coordinate Framework version 3, enhancing it with two flatmaps of the whole isocortex. We used one of these flatmaps to define new annotations of 33 individual barrels and barrel columns that are nonoverlapping and follow the curvature of the cortex, therefore, producing the most accurate atlas of mouse barrel cortex to date. Additionally, we introduced several applications highlighting the utility of laminar coordinate systems for data visualization and data-driven modeling. We provide a free software implementation of our methods for the benefit of the community.

## 1 Introduction

Experimental and computational studies in neuroscience make increasing use of standardized atlases describing the location and extents of brain structures in reference geometric spaces. These atlases are available for a variety of species, including rats (Papp et al., 2014;Paxinos & Watson, 2007), mice (Paxinos & Franklin, 2019;Wang et al., 2020), and humans (Amunts et al., 2020). By using registration methods, it is possible to place data from different origins and across different modalities into the common coordinate space of such an atlas. Data integrated in this way can be represented and analyzed in novel ways (Leergaard & Bjaalie, 2022), and provides the building blocks for data-driven modeling (Reimann et al., 2022).

Unlike traditional printed atlases that consist of a series of labeled outlines of brain slices along one or more cut planes, digital brain atlases represent information as a three-dimensional matrix where each voxel is labeled with the region to which it belongs. Typically, the Cartesian coordinate system defined by the voxel indices is aligned to match the anatomical axes of the brain (transverse, coronal, sagittal). As such, digital brain atlases provide a lookup for the locations of brain regions in global anatomical coordinates.

Although the Cartesian coordinate system is anatomically well defined, it does not necessarily provide the best context in which to describe the shape and structure of particular brain regions. Thus, many regions have been traditionally analyzed and modeled with respect to region-specific coordinate systems that better capture their functional and developmental organization. For example, hippocampus has a complex geometry where a path following its local depth axis tends to be curved, and different paths can have entirely opposite orientations in the different hippocampal subfields (Strange et al., 2014). Since these properties cannot be accounted for by simple linear or affine transforms of the Cartesian coordinate system, hippocampal geometry has been described in terms of long-axis, proximal-distal, and laminar coordinates in human (DeKraker et al., 2018) or longitudinal, transverse, and radial coordinates in rat (Romani et al., 2023). Even thalamic nuclei contain functional gradients that can be expressed as axes of a region-specific coordinate system (Sumser et al., 2017).

Furthermore, as data are recorded over increasingly larger portions of the brain, the existence of continuous region-wide coordinate systems begins to constitute a requirement for data integration and analysis (Gao et al., 2022). Also, the construction of large-scale models of entire brain regions is likely to rely on algorithms that are defined in such coordinate systems adapted to the particular geometry of each region. Therefore, it becomes imperative to provide a reproducible and objective way to define these region-specific coordinate systems.

In the case of layered regions such as the neocortex, it is possible to define a particular kind of coordinate system that considers the curvature of the region as well as the laminar structure, providing a powerful tool to work with these complex geometries. We name this a*laminar coordinate system*and formalize its properties. First, the coordinate system is primarily defined by one*principal axis*of organization whose orientation varies smoothly in space. Second, the local orientation of this principal axis can be determined by the direction along which the layers change at each point in space. Third, the other two axes are locally orthogonal to the principal axis, but otherwise their orientation is of secondary importance.

The two axes orthogonal to the principal axis describe a*flatmap*of the region. The concept of a flatmap bears a resemblance to cartographic projections of the globe (Hahn & Duckworth, 2023), but is more complex, as it projects a three-dimensional structure and not just its surface. The main property of a flatmap is that it allows the seamless mapping of information back and forth between 2D and 3D spaces in a way consistent with the principal axis. It involves a kind of structured dimensionality reduction where information is aggregated in a direction orthogonal to the layers. For example, a circle drawn on the flatmap corresponds to a 3D subvolume similar to a cylinder, whose axis follows the local direction of the principal axis, and where the layer structure is preserved; in the context of the neocortex, such a subvolume would represent a cortical column, if appropriately sized. Conversely, any point inside this column would map back to the circle drawn on the flatmap.

In this manuscript, we introduce a general method to define laminar coordinate systems and flatmaps based on digital brain atlases, and provide a free software implementation for the benefit of the community (see Data and Code Availability). In order to ensure flexibility, and given the spatial discretization of the voxelized inputs, we do not attempt to derive a mathematical description of the laminar coordinate system. Instead, we represent it in the form of*auxiliary atlases*, a set of three-dimensional matrices in the same Cartesian space as the base atlas, containing values for all coordinates at the center of each voxel; additionally, we also define the principal axis vector at each voxel. We call the process of generating auxiliary atlases*atlas enhancement*. Our approach of releasing tools to create and optimize flatmaps based on user specifications sets us apart from previous work that only provided end products, such as the flatmap of mouse isocortex inWang et al. (2020).

While being of general applicability, we present here applications of our method to two rodent atlases. First, we enhance an atlas of rat somatosensory cortex (based onPaxinos & Watson, 2007) with a laminar coordinate system adapted to the geometry of this region. Second, we enhance the Allen Mouse Brain Common Coordinate Framework version 3 (CCFv3;Wang et al., 2020) with two flatmaps of the whole mouse isocortex and use one of them to refine the annotations in the barrel cortex area (SSp-bfd) with individual barrels and associated barrel columns. Similar to a cartographic projection, a flatmap inevitably introduces distortions. Therefore, we also define several metrics that can be used to quantify local and global distortions and assess the quality of the results. Finally, we present some applications of flatmaps for data-driven modeling and data analysis and visualization.

## 2 Methods

We introduce methods to generate the three axes of a laminar coordinate system in the form of auxiliary atlases. We describe the algorithms in detail but leave out the particularities of our implementation; these can be found inSupplementary Material E. Specific values for the various parameters of the algorithms are provided in theSection 3for the use cases presented there.

### 2.1 Generating depth and orientation fields

In order to define the principal axis, we propose an algorithm that generates auxiliary atlases of depth and local orientation. Specifically, depth information at each voxel is provided by a relative depth value (between 0 and 1) and a local thickness (inμm); absolute depth (inμm) can be easily computed as the product of these two. Local orientation is provided by a vector defined at each voxel; this vector is represented by a rotation of the Cartesian Y axis and stored as a rotation matrix.

The input to the algorithm is a voxelized brain atlas with region annotations, that is, each voxel has a label of the brain region to which it belongs. We select a subset of regions of interest, for example, all isocortex regions or somatosensory regions, and use them as the*source volume*for the following steps.

**Fig. 1.**
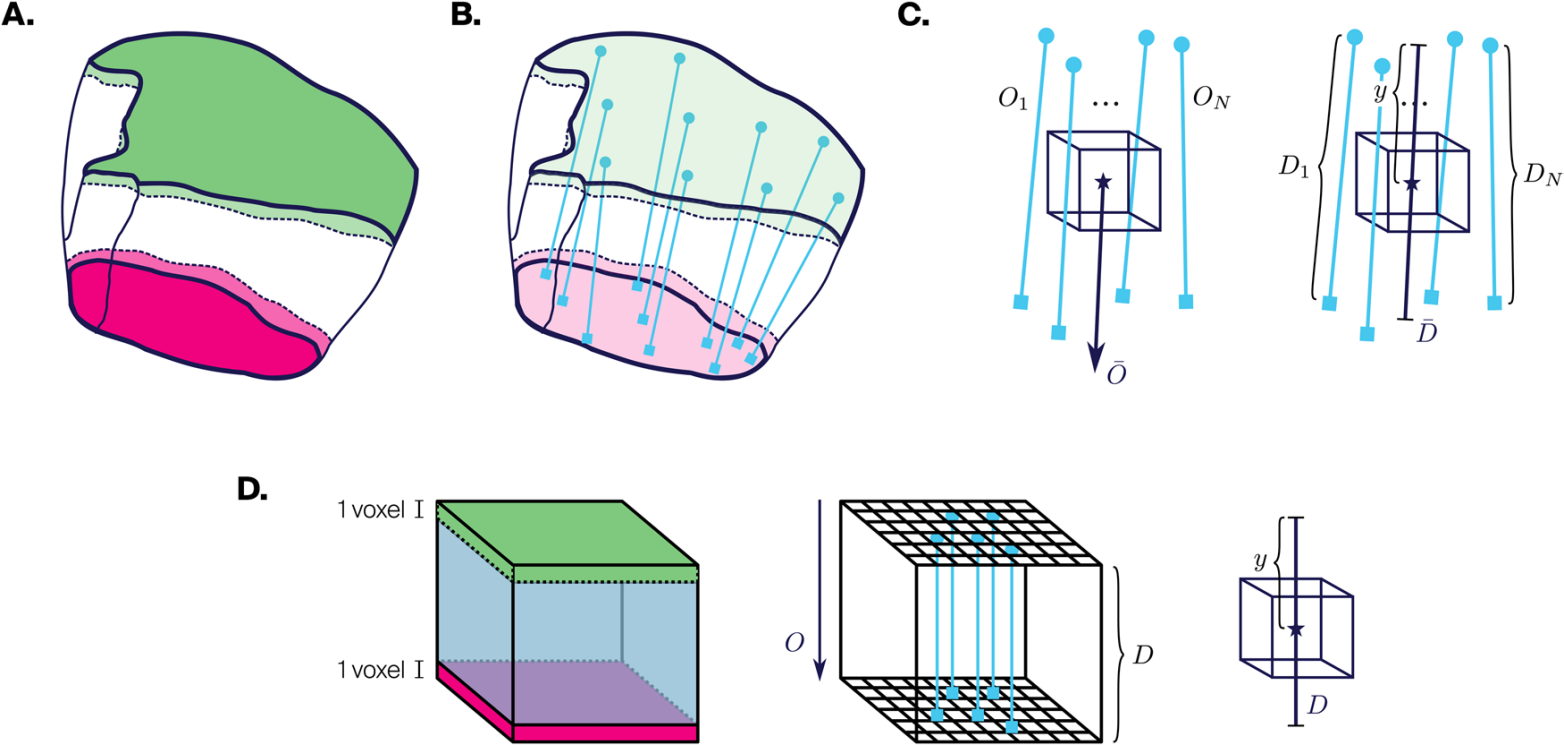
Generating depth and orientation fields. (A) Top (green) and bottom (pink) shells. (B) Shortest line segments (light blue) between pairs of voxels in top and bottom shells. (C) Left: Average direction ofNclosest line segments gives local orientation$\bar{O}$. Right: Average length ofNclosest line segments gives local thickness$\bar{D}$. Ratio of distance from top shellyto local thickness gives local depth$d=\frac{y}{\bar{D}}$. (D) Illustration of these concepts in the simple geometry of a cube. In addition to top (green) and bottom (pink) shells, sides are labeled (light blue). Shortest line segments are all parallel and have the same orientationOand same heightD. There is a single line segment passing through every voxel center.

We begin by marking the top and bottom voxel shells in the source volume. These shells consist of one-voxel thick layers located at the extremes of the source volume (zero depth and maximum depth), which would correspond to the cortical surface and the white matter boundary of the neocortex, respectively (Fig. 1A). Given the complexity of this task in actual brain region geometries (seeFig. 1Dfor an illustration in a simple geometry), it is performed by expert manual annotation. In short, we first consider all voxels that are in contact with the exterior of the source volume, and then we interactively “paint” them (assign them a label) as top or bottom shell; the remaining unlabeled voxels constitute the sides. The result of this process is an auxiliary atlas where each voxel is labeled as exterior, boundary (top, bottom, or sides), or interior. We characterize timing and interannotation variability by considering the annotations from two additional experts, beyond the original used in the results.

Afterward, we find all line segments joining a voxel in the top shell with its closest voxel in the bottom shell, and vice versa (Fig. 1B). We use these line segments to define the local orientation of the principal axis and the local thickness of the source volume (measured along the principal axis) at each voxel (Fig. 1C).

To define the local orientation vector of a voxel, we consider theNclosest line segments to its center and compute their average direction$\bar{O}$. Conventionally, in order to measure depth, we take the sign of the vector pointing toward the bottom shell. To define the local thickness, we take the average length$\bar{D}$of theseNclosest line segments. Finally, to define the local relative depthd, we measure the distanceyfrom the voxel center to the top shell along$\bar{O}$and take the quotient with$\bar{D}$, that is$d=\frac{y}{\bar{D}}$.

The numberNof line segments used per voxel determines to what degree the resulting orientation and depth fields are spatially smoothed. Artifacts in the region annotation atlas and its spatial discretization can lead to sudden jumps in the measurements, making some degree of smoothing necessary; thus,Nshould be chosen depending on voxel resolution and the geometry of the source volume.

### 2.2 Flatmapping

In order to define the other two axes orthogonal to the principal axis in a laminar coordinate system, we introduce a flatmapping algorithm. Intuitively, the process of flatmapping is like squeezing the neocortex by pressing simultaneously from the top and bottom at every point, collapsing all layers into a very thin, but curved sheet of cortex; if we flatten this sheet, and draw coordinate axes on it, we obtain a flatmap. These steps must be done smoothly to preserve*connectedness*, that is, the 3D subvolume associated with a 2D area has a single component, and*continuity*, that is, 3D subvolumes associated with neighboring areas are neighbors themselves, in the result.

The input to the flatmapping algorithm is a definition of the principal axis, along which the source volume is to be flattened. Specifically, we require auxiliary atlases of local orientation and relative depth, as are generated by the algorithm in the previous section. The algorithm is flexible, however, and inputs generated by other methods can be used, as long as they provide the same information.

The flatmapping algorithm has two stages (I, II) which can be performed in parallel and converge into a final stage (III). Since the algorithm involves computing depths and orientations at arbitrary locations and not just voxel centers, we define continuous functions through linear interpolation, giving depthd(x)and orientationO(x)at any pointxinside the source volume.

#### 2.2.1 Stage I: Flat mesh generation

**1. Definition of projection mesh**The first step is choosing a*projection surface*onto which three-dimensional points are projected. This surface is defined as a level set (isosurface) of the relative depth field, that is, the set of all points at the same relative depth$d^{*}$. Note that this surface is only defined as an abstraction, as it cannot be computed exactly in a numerical setting.

What can be generated is a*projection mesh*, a triangular mesh that approximates the projection surface. To this end, first we find all voxels (approximately) at a relative depth of$d^{*}$. We do so by defining two binary masks, one with voxels at a relative depth less than$d^{*}$and the other with the rest of the source volume and the exterior. We then take the voxels at the boundary between these two masks and remove any isolated voxels to have a single connected component.

Then, we apply a surface reconstruction algorithm to generate a triangular mesh from the point cloud of voxel centers. We use the scale-space surface reconstruction algorithm (van Lankveld, 2023), as it gives good results and produces a mesh that contains the original points and thus approximately matches the projection surface. Following inspection of the output, manual fixes can be performed on the input point cloud to improve the reconstructed mesh.

Finally, we increase the number of vertices in the triangular mesh through refinement by subdivision (Geuzaine & Remacle, 2009), that is, introducing new vertices at the midpoints between existing vertices and connecting them to define new triangles. This is needed in order to minimize errors when mapping to mesh vertices (see stage III.1). We apply this procedure one or more times to attain a number of vertices in the mesh that is as close as practically possible to the number of voxels in the source volume.

**2. Flattening of projection mesh**Flattening of the projection mesh is performed using an authalic (area-preserving) texture mapping algorithm (Saboret et al., 2023). There are two versions of this algorithm, a discrete one that is fast and we use for simple meshes, and an iterative one that takes longer but gives better results for complex meshes. These algorithms take as input a triangular mesh with one connected component and a continuous boundary, and as output a triangular mesh that is flat (contained in the unit square) and has the same number of points as the input mesh, in a one-to-one correspondence. We call this flattened triangular mesh the*flat space*andℱis the flattening operator that maps points on the projection mesh to points in flat space. An area-preserving algorithm is chosen to keep the relative sizes of regions in flat space as in the source volume.

An important step in the flattening algorithm is the choice of shape of the flat space. While always contained in the unit square, the border can have any convex shape specified by the user, for example, any convex polygon or convex parametric curve. The simplest choice is to use the unit square itself, which also lends itself well for volume decomposition (seeSection 2.6.1) and computation of flatmap metrics with square pixels (seeSection 2.5), but other shapes can be chosen depending on the use case. Furthermore, the way in which the border of the projection mesh is mapped to the border of the flat space, which determines the orientation of the axes in flat space, can be prescribed by either manually specifying corresponding vertices or by shifting the first vertex along the border.

#### 2.2.2 Stage II: Voxel projection

In this stage we compute a mapping from voxel centers to points on the projection surface. This stage can be performed in parallel to stage I, as it does not require the projection mesh but only the definition of the projection surface (as a level set of the relative depth field).

**Fig. 2.**
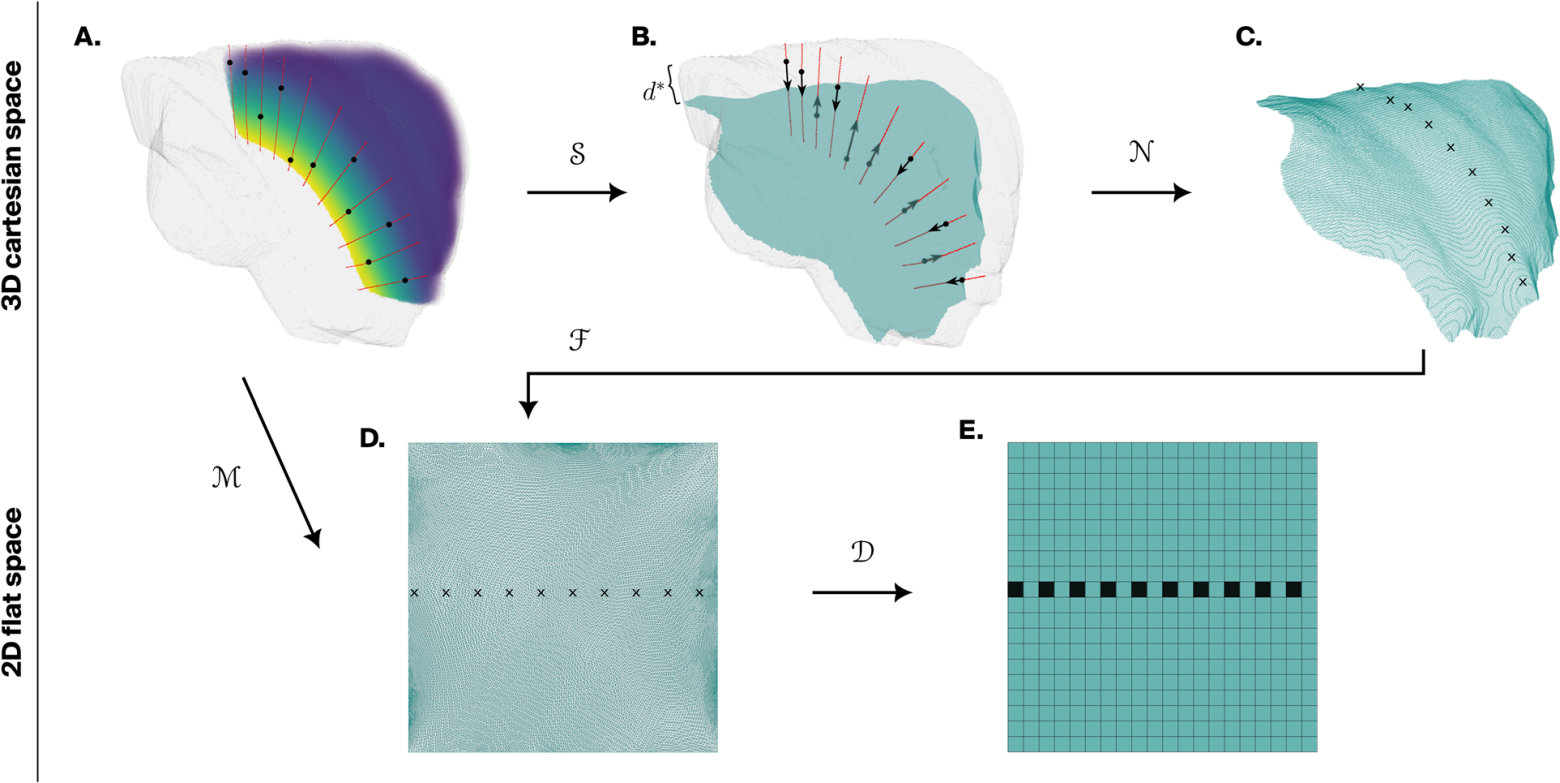
Flatmapping algorithm. (A) Streamlines (red curves) are computed by numerical integration of the orientation field using voxel centers (black dots) as initial positions. The relative depth field is represented as a colored volume ranging from low depths (purple) to high depths (yellow). (B) Voxel centers are mapped to the projection surface at relative depth$d^{\text{*}}$(turquoise) following streamlines (black arrows, operatorS). (C) Points on the projection surface are mapped to vertices (black crosses) on the reconstructed projection mesh (turquoise wireframe) through nearest-neighbor search (operatorN). (D) Authalic (area-preserving) flattening of projection mesh into the unit square (operatorℱ). The composite flatmapping operatorℳ=ℱ(N(S(⋅)))maps 3D voxel centers to 2D flat coordinates. (E) Flatmap discretization by grouping of mesh vertices into pixels of chosen size (operatorD).

1. **Computation of streamlines**For each voxelvin the source volume we generate a*streamline*, a 3D curve that follows the principal axis at every point and connects the top and bottom shells while passing through the voxel center (Fig. 2A, intuitively similar to a radial glial cell). In detail, with the center ofvas initial position, we compute an integral curve of the orientation fieldOnumerically (see details inSupplementary Material E). Using a small enough time step, we first iterate in the forward direction until we reach a relative depth of 1 (with$\epsilon_{1}$tolerance); then, we iterate in the backward direction until we reach a relative depth of 0 (with$\epsilon_{0}$tolerance). Finally, we join these two curve segments and define the streamline associated with voxelvas a linear spline$S_{v}\left(t\right)$, where parametert∈[0,1]maps monotonically to points along the full curve.
2. **Projection of voxel centers**We use the streamline$S_{v}\left(t\right)$to find the projection of voxelvonto the projection surface (Fig. 2B). Intuitively, we move the voxel center along the streamline until reaching the projection surface. Technically, we define$F\left(t\right)=d\left(S_{v}\left(t\right)\right)-d^{*}$and use a root finding algorithm to find$t_{v}^{*}$, the intersection between the streamline and the projection surface, that is, the point on the streamline at relative depth$d^{*}$. We call$S=S_{v}\left(t_{v}^{*}\right)$the operator that maps voxel centers to points on the projection surface.

For some voxels, especially at the edges of the source volume, integration ofOmay not reach relative depths of 0 or 1 before leaving the source volume. This may happen due to noise or discontinuities in the orientation field, or truncation of the geometry. These voxels are not*eligible*for flattening and we discard them, since they may not have a projection (if their streamline never attains depth$d^{*}$), and even if they do, the laminar architecture is not preserved along their streamlines.

#### 2.2.3 Stage III: Flatmap generation

Since our ultimate goal is to define a mapping between voxel centers and points in flat space, in the last stage we combine the outputs from the previous stages, namely, the flattened projection mesh and the set of projections of voxel centers onto the projection surface.

1. **Mapping to mesh vertices**For each projection of a voxel on the projection surface, we use a spatial nearest-neighbor search algorithm (Tangelder & Fabri, 2023) to find the corresponding closest vertex on the projection mesh. We call this operatorN(Fig. 2C).
  The distance to the nearest neighbor represents the approximation error of the projection mesh to the projection surface at that location. We try to keep these errors low by reconstructing the projection mesh from points close to$d^{*}$and using iterative refinement to increase the number of vertices in the mesh (see stage I.1). However, if the errors are consistently large (half the voxel size or more), this may be indicative of a problem in the definition of the projection mesh and it should be revised by cross-checking the value of$d^{*}$used, the quality of mesh reconstruction, etc.
2. **Flatmap generation and discretization**Finally, thanks to the one-to-one correspondence between vertices in the projection mesh and flat space, we map each eligible voxel to its*flat projection*. Mathematically, this is represented by the composite*flatmap*operatorℳ=ℱ∘N∘S. In summary, each voxel centervis moved following its streamline to the projection surface, then to the nearest vertex on the projection mesh, and finally to the corresponding vertex in flat space.

The coordinates of the flat projection are called the*flat coordinates*of a voxel. The highest resolution of a flatmap is determined by the number of vertices in the projection mesh, and we call this the*native resolution*. For certain applications, however, it is desirable to have a regular discretization of the flat space at a lower*pixel resolution*N, meaning that floating-point coordinates in[0,1]are binned into integer indices from0toN−1in both axes (Fig. 2E).

In general, many voxels will have the same flat projection. The set of voxels that map to a given point in flat space, whether at native or pixel resolution, is called the*preimage*of that point, and can be represented by the inverse operator${ℳ}^{-1}$. Pixels with empty preimages, that is, that no voxels map to, may be undesirable depending on the application and can be avoided by a proper choice of pixel resolution.

### 2.3 Distance and area measurements in flat space

Given that the flattening algorithm always maps the projection mesh into the unit square, flat coordinates are normalized to[0,1]and distance units are lost. In order to recover distance units in flat space, we compute preimage radius at a given pixel resolution (seeSection 2.5.3) and then establish a linear fit between mean preimage radius (inμm) and pixel size. The slope of the linear fit can be used as a proportionality factor between normalized distances in flat space and real distances in Cartesian space. This definition assumes isotropy in flat space, which may or may not be achieved depending on the aspect ratio of the projection mesh.

To measure areas in flat space, since the flattening algorithm is area preserving, it suffices to use the original area of the projection mesh as a proportionality factor.

### 2.4 Characterization of depth and orientation fields

In order to quantify their smoothness, we compute the Laplacian of the depth field and the divergence of the orientation field at every voxel, and check how close these quantities are to 0. That is, we measure how close the depth and orientation fields are to being a homogeneous solution to the heat equation and its gradient, respectively. We compute statistics of these quantities over all interior voxels (not in the top, bottom, or sides), to reduce bias due to discrete numerical derivative artifacts at the boundaries of the source volume.

### 2.5 Flatmap characterization

As mentioned before, important properties of a flatmap are continuity, connectedness, and mapping of 2D areas to 3D subvolumes with preserved layer structure. We define some metrics to characterize how close the output of our flatmapping algorithm is to having these desired properties. Some are global or per-voxel metrics, but otherwise they are defined for each pixel at a given pixel resolution. These per-pixel metrics characterize the structure of the preimages and can be further analyzed in terms of statistics computed over all pixels and across pixel resolutions.

#### 2.5.1 Global metrics

##### 2.5.1.1 Coverage

Fraction of eligible voxels in the source volume, that is, number of voxels having a flat projection over total number of voxels. Under normal circumstances, this value is expected to be close to 1, with some noneligible voxels at the edges of the source volume. A value much lower than 1 may be indicative of issues with the orientation field or the integration of streamlines.

##### 2.5.1.2 Usage fraction

Fraction of pixels in flat space with a nonempty preimage, that is, with at least one voxel mapping to them, computed at some pixel resolution. At low resolution, the usage fraction is almost certainly equal to 1, whereas at higher resolutions, holes eventually appear and the usage fraction becomes lower than 1. The optimal pixel resolution depends on the application, and this metric can help in choosing it. At native resolution, the usage fraction is the ratio of the number of vertices with nonempty preimages to the total number of vertices in the mesh.

#### 2.5.2 Per-voxel metrics

##### 2.5.2.1 Orthogonality

Degree to which the principal axis is orthogonal to the flatmap axes at each voxel. We compute this by first taking the gradient of both flat coordinates considered as scalar fields in Cartesian space. Then, we compute the cross product of these two gradients and choose the sense toward the bottom shell. Finally, we measure the cosine of the angle between this vector and the orientation vector by taking the dot product after normalization. A value of 1 indicates that the principal axis is locally orthogonal to the flatmap, whereas a lower value indicates some degree of nonorthogonality. If this quantity is negative, it means the cross product points in the opposite direction as the orientation vector, and a sign flip is needed for proper comparison.

#### 2.5.3 Per-pixel metrics

##### 2.5.3.1 Preimage size

**Fig. 3.**
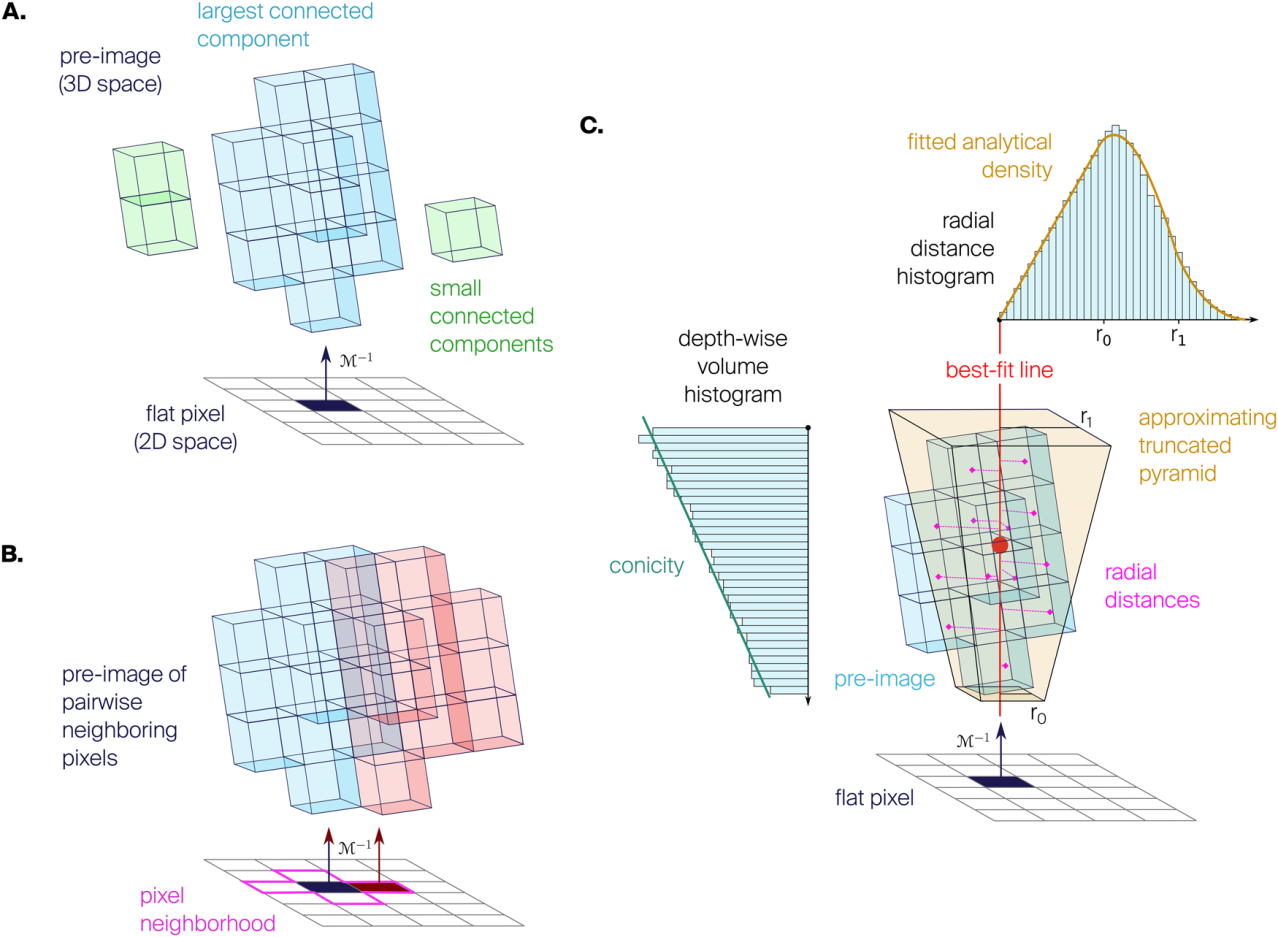
Flatmap characterization. (A) Preimage size and connectedness. The preimage may consist of one (blue) or more (green) connected components. (B) Preimage continuity for a pair of neighboring pixels (red, blue). The metric is evaluated over the four pairs in the pixel’s neighborhood (magenta). (C) Preimage radius is computed using the radii of the best approximating truncated square pyramid, determined by fitting the analytical density to the histogram of radial distances from the best-fit line. Preimage conicity is the slope of a linear fit to the volume histogram as a function of depth.

Number of eligible voxels in the preimage of a pixel (Fig. 3A).

##### 2.5.3.2 Preimage size uniformity

Percentage of eligible voxels in the preimage of a pixel, relative to the number expected from pixel area alone if voxels were distributed uniformly throughout. In the ideal case, this metric would reflect the purely geometrical variations in thickness of the source volume, as this would determine how many voxels project to a pixel while following streamlines locally. However, noise and artifacts in the orientation field can lead to off-site streamlines that artificially increase the preimage size at locations far from the neighborhood of a voxel, leading to artifacts visible as nonuniformities in preimage size.

##### 2.5.3.3 Preimage connectedness

Ratio of the number of voxels in the largest connected component of the preimage of a pixel to the number of voxels in the entire preimage (Fig. 3A). We consider two voxels to be connected if one is inside the 3 x 3 x 3 neighborhood centered on the other (26 adjacent voxels). This metric quantifies how much the preimage consists of a single chunk versus multiple separate pieces. By taking the ratio of the largest component to the total, instead of the number of connected components, we avoid bias due to possibly one large and many small components (even single voxels). At native or high pixel resolutions, preimages are expected to be disconnected since the noise in the geometry and the orientation field is more evident than at lower resolutions.

##### 2.5.3.4 Pairwise preimage continuity

Same as preimage connectedness, but computed for the combined preimage of a pair of adjacent pixels. Given there are four adjacent pixels in a pixel’s neighborhood, we take the minimum value (worst case) over all four pairs (Fig. 3B). This metric provides a measure of local continuity in the sense that moving from one pixel to the next in flat space should represent a corresponding movement between contiguous places in Cartesian space. This is important, as otherwise it would not be possible to relate close points in Cartesian space to close points in flat space. A discontinuous flatmap would be indicative of issues with the orientation field, as streamlines should vary smoothly and not cross or deviate from their neighbors.

##### 2.5.3.5 Preimage conicity

Slope of a line fitted to the depth-wise volume histogram of the preimage of a pixel (Fig. 3C). When conicity is nonzero, the preimage has relatively more or less volume at higher depths (for positive/negative values, respectively), and thus resembles a cone. When conicity is 0, the preimage has a uniform volume distribution along depth, resembling a cylinder.

##### 2.5.3.6 Preimage radius

Extent of the preimage of a pixel in the directions orthogonal to its main axis. To give some intuition, if the preimage was a cylinder, this would be its radius. However, for a square pixel the preimage looks more like a truncated square pyramid (due to conicity), so we require a way to measure the extent of such an object. We approximate it in three steps (Fig. 3C). First, the center of the preimage is calculated as the centroid of its voxels. The main axis of the preimage is defined as a line passing through its center and having the direction that minimizes the sum of square distances to all of its voxels, that is, the first principal component of the voxels’ coordinates. Then, the radial distance from the main axis to each voxelr(v)is calculated, and its histogram is fitted using the analytical formula for a truncated square pyramid of radii$r_{0}$and$r_{1}$(seeSupplementary Material F). Finally, the preimage radius is defined as the mean of the fitted radii$\left(r_{0}+r_{1}\right)/2$.

### 2.6 Applications of flatmaps

#### 2.6.1 Volume decomposition

The flatmap can be used to decompose the source volume into uniform and nonoverlapping subvolumes that preserve the laminar structure, for example, each sampling the complete stack of cortical layers in the case of the neocortex.

In general, to define a subvolume, we first draw a shape in flat space (preferably at native resolution) and take all points that are inside this shape. Then, we take the preimages of all these points and consider their union, that is, the set of all voxels that under the flatmap operator project to this flat shape. The resulting set of voxels describes a 3D volume that “follows” the streamlines and keeps the laminar structure intact.

Now, if we draw a tiling in flat space (e.g., square or hexagonal grid) and consider the subvolumes corresponding to all elements of the tiling, we obtain a decomposition of the entire source volume. These subvolumes are nonoverlapping as long as the flat shapes are nonoverlapping, as is the case for a plane tiling, and their properties can be characterized using the metrics described above (taking “per-pixel” to mean “per-element of the tiling”).

#### 2.6.2 Flat views of three-dimensional data

Any quantity defined for the voxels in the source volume, such as region annotations or injection densities, can be assigned to their flat projections, resulting in a*flat view*(2D representation of 3D data) of such a quantity. Because of this decrease in dimensionality, some aggregation function must be applied to assign the value at a flat point using the combined values of the voxels in its preimage. Once we have the aggregated values, we can use a color palette to obtain a heatmap of the quantity of interest.

We colloquially call*flatmap*to the flat view of region annotations, and we use the mode (most common value) as the aggregation function. For flat views of other volumetric datasets, we typically use the mean or maximum (similar to a maximum intensity projection) as aggregation function.

A script to generate flat views of any 3D dataset registered to the same atlas used to produce the flatmap is provided in the software repository (see Data and Code Availability) together with instructions on how to use it.

#### 2.6.3 Annotation of barrel columns in the mouse isocortex

A variant of the volume decomposition procedure can be used to generate annotations of barrel columns in the mouse isocortex. For this use case, the shapes drawn in flat space are the outlines of the individual barrels, generated as follows.

Firstly, barrel annotations were extracted based on the “average template” two-photon tomography dataset part of CCFv3 (Wang et al., 2020), where the anatomical features of the barrel and septum separation are visually apparent.

Secondly, we performed a semiautomatic segmentation in ITK-SNAP using the Active Contour Snake algorithm (Yushkevich et al., 2016), and obtained volumes for 33 ellipsoidal barrels in the left hemisphere. We annotated the 33 barrels as separate structures with labels of their corresponding rows and arcs. Segmentation of additional barrels was unsuccessful due to the limited signal-to-noise ratio of the average template dataset.

Thirdly, the new annotations were projected onto flat space using the flatmap of the mouse isocortex (Fig. 6A). We used the flat positions to further refine the annotations by reassigning mislabeled voxels to the label of their nearest neighbor.

Lastly, using the final annotations, we computed the convex hull of all flat positions belonging to a barrel and took the preimage of all points inside it. This way we obtained cylinder-shaped barrels and barrel columns. The originally ellipsoidal barrels were extended to span the complete height of layer 4 (Fig. 6B).

#### 2.6.4 Flat views of long-range axons in the mouse isocortex

Any reconstructed morphology with points inside the source volume can be visualized in flat space. This is done by simply taking the flat projection of the points representing the soma and neurites, and plotting them over the flatmap.

In particular, for visualization of long-range axons in the mouse isocortex, we downloaded in bulk the latest MouseLight data release fromml-neuronbrowser.janelia.org(dated February 28, 2022) and worked with JSON morphologies in the CCFv3 space. We then plotted the flat projections of the soma and axon points on top of our flatmap of mouse isocortex for both hemispheres (seeFig. 7).

## 3 Results

### 3.1 Enhancement of rat somatosensory cortex atlas

**Fig. 4.**
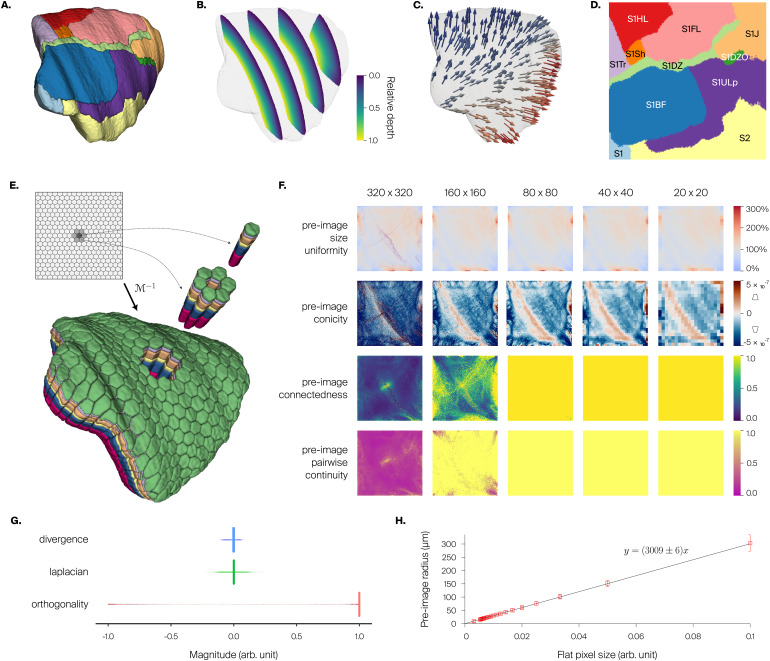
Enhancement of rat somatosensory cortex atlas. (A) 3D atlas with region annotations for one hemisphere. (B) Relative depth field, represented as sections with color map giving relative cortical depth. (C) Orientation field, represented as small 3D arrows (sample). (D) Flatmap of somatosensory regions in one hemisphere (same colors as in A), with labels. (E) Decomposition of atlas volume into hexagonal subvolumes using the inverse flatmap operator${ℳ}^{-1}$. Preservation of layer structure (in colors, from L1 in green to L6 in magenta) is evident in hexagonal columns shown separated from the whole. Inset shows hexagonal grid drawn on flat space, with highlight of central hexagon (dark gray) and surrounding hexagons (light gray). (F) Quality metrics as a function of pixel resolution. For preimage size uniformity, the color bar indicates the proportion of voxels mapped to a pixel compared with that expected from pixel area alone in the perfectly uniform case; values at or above 300% are colored the same. For preimage conicity, the small diagrams next to the color bar give an intuition on the meaning of positive or negative conicity. We show power-of-two pixel resolutions to have nice subsampling in plots. In general, degradation is visible near the edges. (G) Distributions of values of per-voxel metrics: divergence of orientation field, Laplacian of relative depth field, and orthogonality between principal axis and flatmap axes. (H) Distance metric obtained from linear fit of flat pixel size versus preimage radius (mean and std. dev.).

We applied our methods to an atlas of rat somatosensory cortex with 10 annotated regions (Fig. 4A), based on a well-known printed atlas of the rat brain (Paxinos & Watson, 2007). Details of the creation of this atlas and a description of the region labels can be found inSupplementary Material A.

Marking of the top and bottom shells was done by manual “painting” of voxels in about 10 minutes (3% interannotation variability betweenn=3expert annotators), owing to the simple geometry of the somatosensory cortex. Afterward, we generated the relative depth field (Fig. 4B) and orientation field (Fig. 4C) by samplingN=1,000nearest line segments at each voxel.

For flatmapping, the source volume consists of all somatosensory regions, encompassing1,314,885voxels. The projection surface is taken as the isosurface of the relative depth field at$d^{*}=0.5$. The reconstructed projection mesh has23,314vertices and an area of40.606mm^2^, and it was refined three successive times into1,492,096vertices. The resulting flatmap (Fig. 4D) shows clearly delineated regions. It has a usage fraction at native resolution of0.581with a mean preimage size of1.5voxels, and the coverage is0.988with only15,677noneligible voxels.

We show a decomposition of the source volume into hexagonal subvolumes (seeSection 2.6.1;Fig. 4E). Note how the hexagonal grid defined in flat space is well transferred to Cartesian space. Furthermore, the subvolumes are nonoverlapping and have an intact layer structure, resembling cortical columns.

We investigated the impact of pixel resolution on the properties of preimages by computing a number of metrics (seeSection 2.5;Fig. 4F). We show resolutions from320×320pixels, which is closer to native resolution and is better for drawing on flat space, to20×20pixels, which is close to the resolution at which we decompose the source volume, for example, into hexagonal columns as above. We found the resulting preimage sizes to be relatively uniform at all resolutions, indicating that different parts of the flat space are comparable in how they sample the Cartesian space. However, at a resolution of320×320pixels, the remaining variability still resulted in a number of empty preimages, that is, holes. Preimage conicity was noisier at high resolutions, probably resulting from the uneven surface structure of the source volume (Fig. 4Avs. 4F, second row). At resolutions below80×80pixels, this property was smoother and more uniform. Preimage connectedness and continuity were largely ensured at resolutions of80×80or lower, but fell apart for higher resolutions. For all metrics, the locations of largest nonuniformity were at the edges of the geometry.

Per-voxel metrics show good results (Fig. 4G). Computing the Laplacian of the relative depth field as a measure of smoothness shows good results with a very narrow distribution symmetrical around 0 and median value of0.0003(0.003interquartile range (IQR)). This value is small compared with the range[0,1]of the relative depth field. Computing the divergence of the orientation field as a measure of smoothness also shows good results with a slightly wide distribution symmetrical around 0 and median value of0.0(0.013IQR). We also compute orthogonality of the local orientation vector to the local flatmap axes, obtaining good results with a very sharp distribution near 1 and median value of0.999(0.001IQR). This demonstrates that the decomposition into one principal axis and two axes orthogonal to it was successful.

Finally, we obtained a linear fit of flat pixel size versus preimage radius (Fig. 4H), leading to a distance metric in flat space of3,009±6μm per flat coordinate unit.

### 3.2 Enhancement of mouse isocortex atlas

**Fig. 5.**
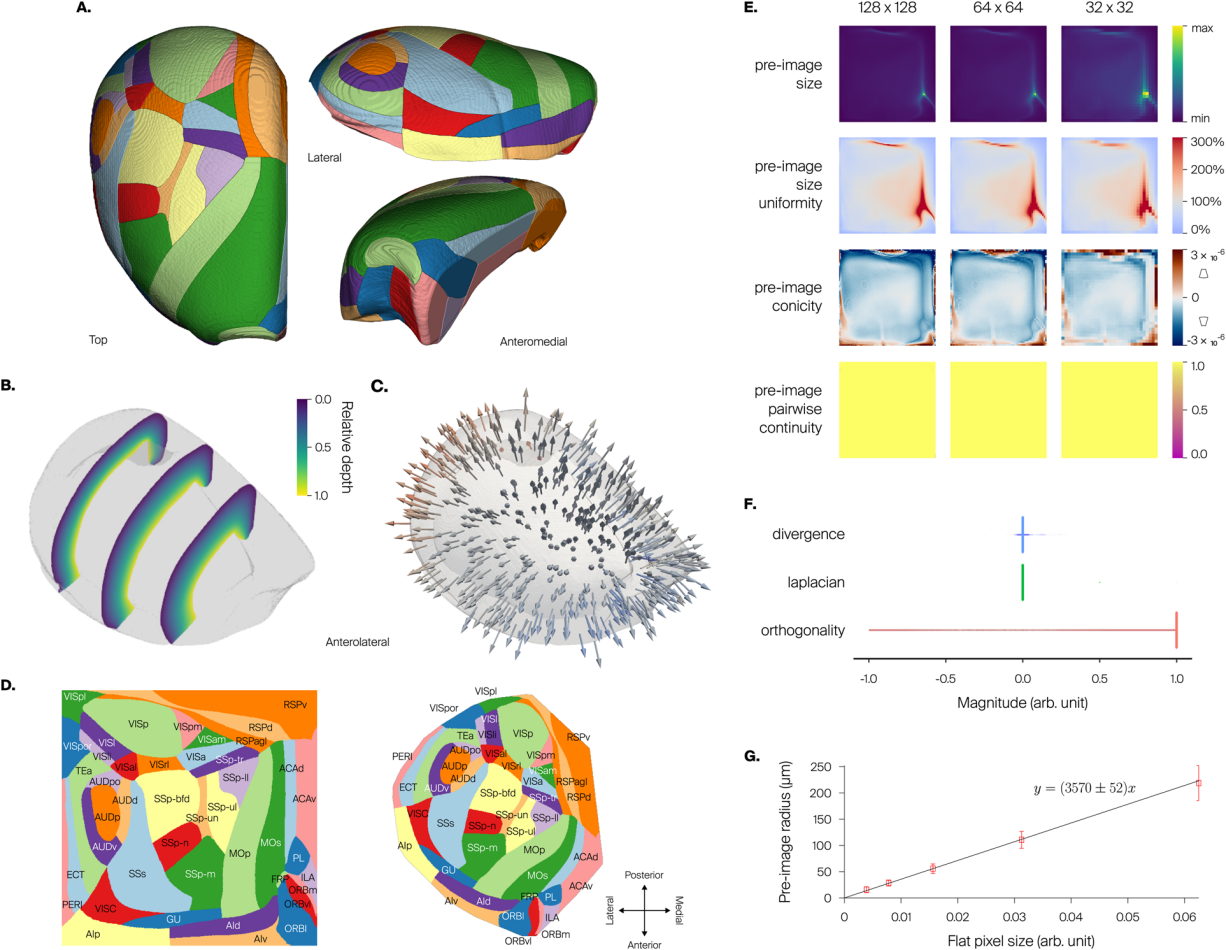
Enhancement of mouse isocortex atlas. (A) 3D atlas with region annotations for one hemisphere. We show top, lateral, and anteromedial aspects of the isocortex for anatomical reference. (B) Solution to Laplace’s equation as depth field, represented as slices with color map giving relative cortical depth in an anterolateral view. (C) Gradient of the solution to Laplace equation as orientation field, represented as small 3D arrows in an anterolateral view. (D) Flatmaps of all isocortex regions in one hemisphere (same colors as in A), with labels. Left: square border. Right: border similar to CCFv3 flatmap, with anatomical axes. (E) Quality metrics as a function of pixel resolution. For preimage size, the color bar indicates the range of values present at each pixel resolution, and cannot be quantitatively compared across resolutions. For preimage size uniformity, the color bar indicates the proportion of voxels mapped to a pixel compared with that expected from the pixel area alone in the perfectly uniform case; values at or above 300% are colored the same. For preimage conicity, the small diagrams next to the color bar give an intuition on the meaning of positive or negative conicity. We show power-of-two resolutions to have nice subsampling in plots. “Hotspots” in preimage size are visible in the frontal pole and other regions of high cortical curvature. (F) Distributions of values of per-voxel metrics: divergence of orientation field, Laplacian of relative depth field, and orthogonality between the principal axis and flatmap axes. (G) Distance metric obtained from a linear fit of flat pixel size versus preimage radius.

We use existing datasets from the Allen Mouse Brain Common Coordinate Framework (CCFv3) at 10μm resolution to generate a flatmap of the entire mouse isocortex (see Data and Code Availability). We consider all 43 regions under “Isocortex” in a single hemisphere as the source volume (Fig. 5A). The availability of top, bottom, and side shells in the CCFv3 would have allowed us to derive the relative depth and orientation fields with our method. However, we chose to use the already available solution to the Laplace equation as depth field (Fig. 5B) and its numerical gradient as orientation field (Fig. 5C), to showcase the flexibility of our method in working with external datasets obtained using other methods. When computing the gradient of the solution, we extended it one voxel toward the outside to reduce edge artifacts in the numerical derivatives.

We take as projection surface the isosurface of the Laplacian solution at$d^{*}=0.5$. The reconstructed projection mesh has437,186vertices and an area of51.652mm^2^, and it was refined three successive times into27,942,965vertices, to approximate the61,945,750voxels in the source volume. To showcase once more the flexibility of our method, we produce two flatmaps targeting different use cases, one with a square border for volume decomposition (seeSection 3.3), and the other with a similar border as the CCFv3 flatmap (from now on referred to as “shape-match” flatmap), in order to perform a comparison with it and to provide a more familiar shape for data visualization.

The resulting flatmaps show clearly delineated regions (Fig. 5D). They both have a usage fraction at native resolution of0.833with a mean preimage size of2.7voxels, and a coverage of0.997with only213,025noneligible voxels, since these metrics do not depend on the shape of the border. The shape-match flatmap represents the same regions and in the same continuous manner as the square border flatmap, but has a more intuitive curved shape and better aligned anatomical axes, similar to the CCFv3 flatmap (for a more in-depth, side-to-side comparison seeSupplementary Material C).

**Fig. 6.**
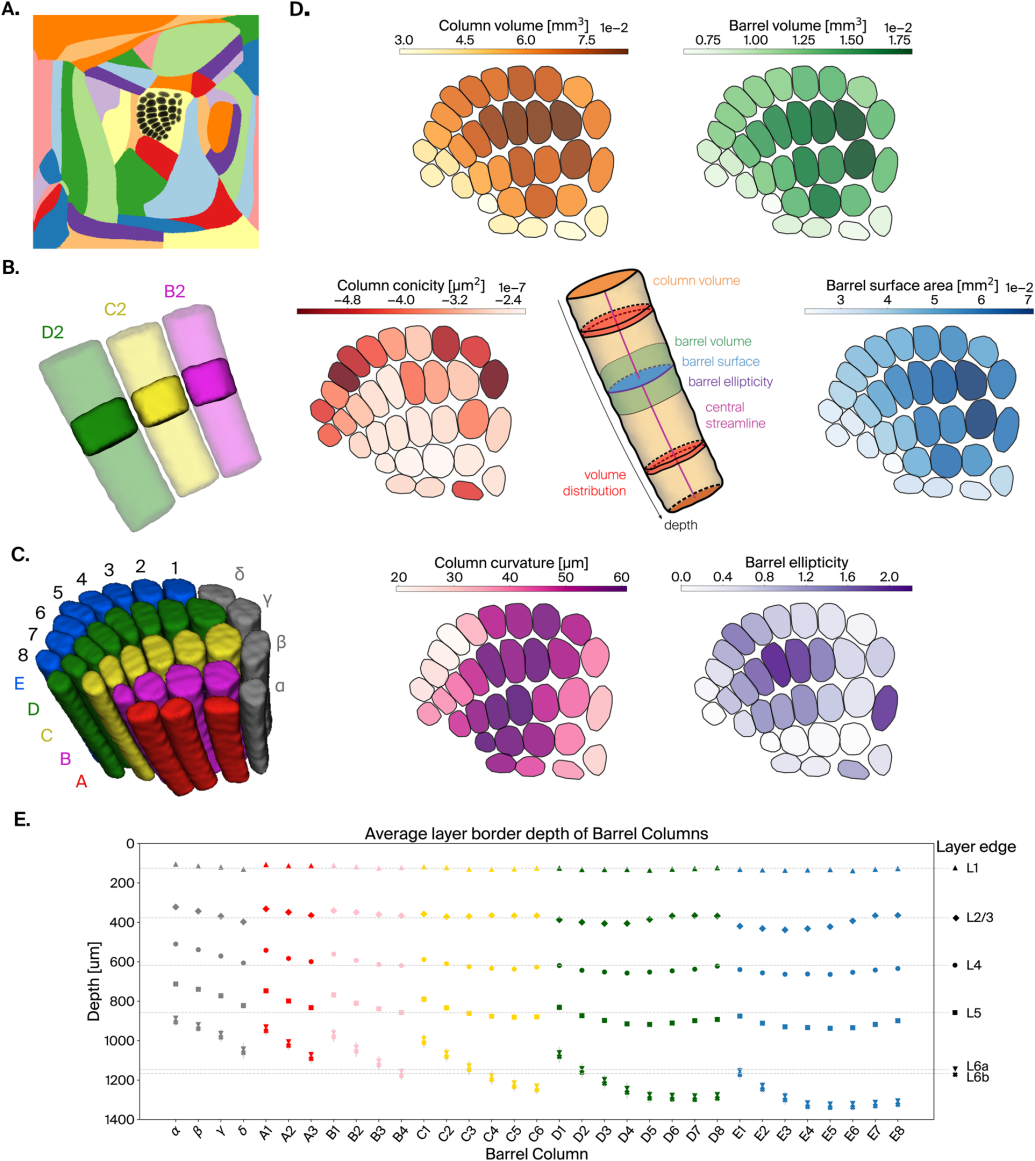
Enhancement of mouse barrel cortex atlas. (A) Flat projections of annotated barrel voxels (seeSection 2, black) in SSp-bfd, shown in the context of the full mouse isocortex. (B) Barrel volumes of 2-row (solid) and their corresponding columns, defined as the preimages of their flat projections (semitransparent). (C) Extracted barrel columns for all annotated barrels, barrel rows represented by the same color. (D) Barrel and barrel columns metrics. The center schematic illustrates how they are defined. Followed by heatmaps of their values plotted on flat projections of barrels. The metrics are (clockwise):*Barrel volume*, shown as an outline (green) in the schematic.*Barrel surface area*, shown as a cross-section (blue) in the schematic.*Barrel ellipticity*, computed as the ratio of major to minor axis of an ellipse fitted to the flat projection of a barrel, represented by the perimeter (purple) in the schematic.*Column volume*, shown as a volume (orange) in the schematic.*Column curvature*, defined as maximal displacement from a straight line fitted to the barrel streamline, shown as a line (pink) in the schematic.*Column conicity*, computed as the slope of a linear fit to the depth-wise volume histogram, represented by two volume bins (red) in the schematic. (E) Average layer border depth for all layers in each of the barrel columns. Gray lines indicate mean layer depths across all barrel columns.

In terms of preimage properties of the square border flatmap (Fig. 5E, for the shape-match flatmap seeSupplementary Fig. 6A), we found that it was almost completely connected and continuous, without holes, at resolutions up to256×256pixels (not shown for simplicity). Higher pixel resolutions could not be investigated due to computational costs stemming from the high resolution (10μm) of the input atlas, but based on the trends of the metrics computed for the rat flatmap above, a breakdown of connectedness would be expected at a resolution of1,024×1,024pixels, and the appearance of holes at a resolution of2,048×2,048pixels. Preimage conicity was smoother than for rat at high resolutions, reflecting the smoothness of the input atlas. One noteworthy feature visible in the preimage size and uniformity at all pixel resolutions is the presence of “hotspots” in the frontal pole and in medial regions (compare withFig. S2BinWang et al., 2020). These are places of high curvature in the cortex, where due to convergence of orientation vectors, many voxels are mapped to the same pixel.

The per-voxel metrics showed excellent results (Fig. 5F, for the shape-match flatmap, seeSupplementary Fig. 6B). As expected, the Laplacian of the depth field (itself a solution to Laplace equation) was very close to 0, with a median of$1.0\times 10^{-5}$($2\times 10^{-5}$IQR), and the divergence of the orientation field was correspondingly also very close to 0, with a median of$1.1\times 10^{-5}$($2\times 10^{-5}$IQR). Orthogonality showed once more a successful decomposition into a principal axis and two orthogonal axes, with a median of0.999(0.0013IQR).

Finally, we obtained a distance metric in flat space of3,570±52μm per flat coordinate unit (Fig. 5G, for the shape-match flatmap, seeSupplementary Fig. 6C).

**Fig. 7.**
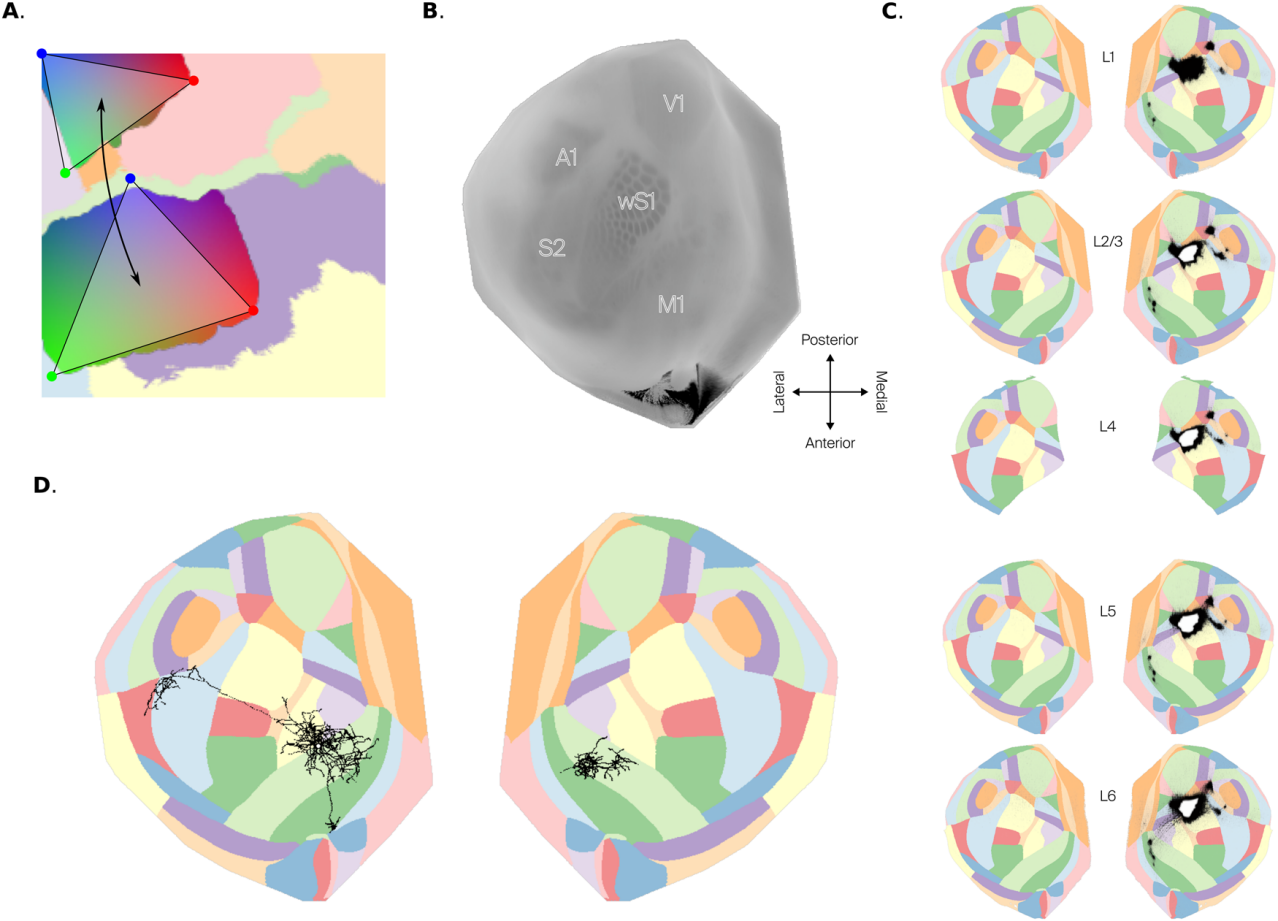
Applications of flatmaps. (A) Topographical mapping between two rat somatosensory regions. Colors represent barycentric coordinates of triangles contained in each region (red, green, and blue vertices). Locations with the same color connect to each other. (B) Flat view of the CCFv3 average template of mouse isocortex. (C) Flat views of injection (white) and projection density (black) across cortical layers in both hemispheres of mouse isocortex. Per-layer maps are stacked, and correspond to L1, L2/3, L4, L5, and L6. (D) Flat view of long-range axons (black points) of a neuron with soma (white dot) in primary motor cortex (MOp) of the left hemisphere, near the border with lower limb primary somatosensory cortex (SSp-ll).

Since the CCFv3 atlas is perfectly symmetrical with respect to the midline, we extended our flatmaps to both hemispheres by mirroring and adjusting theXflat coordinate to the range[0,1]in the right hemisphere and[1,2]in the left hemisphere. We used the shape-match two-hemisphere flatmap for data visualization (Fig. 7C, D).

### 3.3 Enhancement of mouse barrel cortex atlas

We segmented well-defined barrels from the average template dataset in CCFv3 (Wang et al., 2020) and produced an annotation of the posterior medial barrel field, with 33 individually labeled barrels (Simons & Woolsey, 1979;Welker & Woolsey, 1974). We then used an approach consisting of projecting the barrel annotations to flat space, processing them, and then taking their preimages (seeSection 2.6.3), using the square border flatmap (Fig. 6A), to produce annotations of barrel columns in the CCFv3 reference coordinate space at 10μm resolution (Fig. 6B).

Our efforts allowed us to derive annotations with the following properties: barrels are cylindrical, span the full height of layer 4 and do not overlap, and barrel columns follow the curvature of the cortex. Thanks to these properties, where neighboring barrels are separated by the septum, the associated barrel columns are also separated; where barrels contact each other, their associated barrel columns are also in contact, but never overlap throughout the entire depth (Fig. 6B, C).

To further characterize the geometry of the new annotations, we computed a number of anatomical metrics (Fig. 6D). For the surface of each barrel, we considered the convex hull of the flat projections of the barrel voxels and calculated their area as previously described (seeSection 2.3). To characterize the shape of the barrels, we measure their ellipticity, computed as the ratio of major to minor axis of a confidence ellipsoid fitted to the flat projection of a barrel. The resulting surfaces have an elongated shape, in some cases twice as wide along an arc than along a row, in line withWelker and Woolsey (1974). Furthermore, barrels within a row are very close to each other, often touch, but do not overlap, while big septal areas can be observed between the rows.

We then calculated the volumes of barrels and barrel columns, based on the voxel count and voxel size. In terms of volume and surface area, barrels were generally smaller toward the periphery, with barrel D1 being the largest. Column volume was strongly, but not completely, dependent on barrel surface area. An additional factor was the increase of cortical thickness from row A to row E.

Additionally, we characterized how much the columns deviate from right cylinders, measuring their*conicity*and*curvature*. The former is defined as the slope of a linear fit to the depth-wise volume histogram (seeSection 2.5.3). The latter metric is defined as the maximal displacement from a straight line fitted to the barrel streamline, measured at the top and bottom of the barrel column. Intuitively, we determine how much the barrel column’s streamline deviates from a straight line. For example, from layer 4, the C2 barrel curves 50μm toward the C1 barrel column compared with a corresponding representation as a right cylinder. As a result of the global curvature of the cortical volume, columns were wider at the top than at the bottom. For example, the radius of the C2 barrel column narrowed by 32μm over the entire depth. The strength of this effect was found to be independent of column curvature.

Finally, we measured the location of layer borders along depth in all barrel columns (Fig. 6E). We found that barrel columns increase in height from rows A to E. We also observed a pattern of increasing height with the arc number. However, relative layer depths are largely preserved across barrel columns.

### 3.4 Applications of flatmaps

The flatmaps obtained in the previous section have multiple applications, a few of which we highlight here.

#### 3.4.1 Topographical mapping of long-range connectivity

A flat view of region annotations can be used to visualize and describe the topographical mapping of connections between cortical areas. While the spatial targeting of corticocortical connections is often expressed in terms of laminar synapse profiles (i.e., specificity along an axis orthogonal to layer boundaries), axons from individual neurons also target specific locations along the other two axes, and this can be expressed as a topographical mapping between points in flat space. In our flatmap of rat somatosensory cortex, we parameterized the topographic mapping by using triangles defining local barycentric coordinates per region, and then considering pairs of triangles so that neurons at a given location in one triangle predominately project to the corresponding point on the other triangle (Fig. 7A).

#### 3.4.2 Volumetric data visualization

A flatmap allows us to visualize volumetric data in 2D, as long as the data are registered to the same brain atlas that was flatmapped. We provide a script to do this and instructions in the code repository (see Data and Code Availability). We present here two examples obtained using our shape-match flatmap of mouse isocortex.

We show a flat view (Fig. 7B) of the average template of the adult mouse brain in CCFv3 (Wang et al., 2020). This dataset was constructed by interpolation of tissue autofluorescence data from high-resolution serial two-photon tomography images of 1,675 young adult mouse brains. The flat view was obtained using the maximum as aggregation function, resulting in a flat maximum intensity projection (MIP). Indeed, the resulting image looks similar to a stained and mechanically flattened tangential section of cortex (compareFig. 7Bwith Fig. 1 ofGămănuţ et al., 2018).

We also show connectivity data for a single experiment (ID 126907302) from the Allen Mouse Connectivity Atlas (Oh et al., 2014). Specifically, we show the injection and projection densities of connections originating mainly from the SSp-bfd region, but with slight injection density in the rostrolateral visual area (VISrl). In order to visualize information with laminar specificity, we split the flat view by layer (Fig. 7C). In the images we can see that the strongest cortical targets were parts of the ipsilateral secondary somatosensory and motor areas, the dorsal auditory area (AUDd), and the neighboring anterolateral (VISal) and anterior (VISa) visual areas. Weaker targets, which suffer from high noise, were the remaining visual and auditory regions, and some retrosplenial, temporal, and orbital areas. Other prefrontal and anteromedial areas, including most of primary somatosensory cortex, were largely avoided. Layer wise, we observe that both injection and projection densities spread to all layers, but with slightly higher projection density in infragranular layers.

#### 3.4.3 Long-range axon visualization

We used our flatmap of mouse isocortex to visualize the anatomy and targeting of long-range axons, using data from Janelia MouseLight (mouselight.janelia.org;Economo et al., 2016). Specifically, we show an exemplary mouse axon (ID AA0002) from a cell with soma in the primary motor cortex (MOp) near the border with primary somatosensory lower limb region (SSp-ll). Our approach depicts the targeting and branching structure of all axon collaterals in a single plot. Furthermore, it visualizes the horizontal targeting of axons independently from the vertical one, that is, independently from their laminar termination profiles. In this flat view (Fig. 7D), we can observe branches innervating nearby parts of SSp-ll, as well as ipsilateral secondary somatosensory and motor regions with high specificity. A single branch extends ipsilaterally to secondary somatosensory cortex, while other branches innervate motor regions in the contralateral hemisphere. As the flatmap covers only the isocortex, parts of the axon leaving this part of the brain are not visible. Therefore, the contralateral innervating parts appear separated from the rest and branches innervating subcortical structures are not represented at all.

## 4 Discussion

We have demonstrated a method to enhance brain atlases with three-dimensional coordinate systems adapted to the geometry of layered brain regions. These*laminar coordinate systems*consist of auxiliary atlases describing the principal axis (local relative depth and orientation) and flatmap (other two horizontal axes) of the region of interest. Furthermore, we have defined a set of metrics to characterize the quality of flatmaps, and introduced several applications highlighting the utility of laminar coordinate systems for data visualization and data-driven modeling.

In particular, we applied our method to two rodent atlases. First, to an atlas of rat somatosensory cortex based onPaxinos and Watson (2007)(seeSupplementary Material A), enhancing it with a laminar coordinate system adapted to the geometry of this region. Second, to the well-known Allen Mouse Brain Common Coordinate Framework (Wang et al., 2020), enhancing it with two different flatmaps of the whole isocortex. We used one flatmap to define new annotations of 33 individual barrels and barrel columns that are nonoverlapping and follow the curvature of the cortex, therefore, producing the most accurate atlas of mouse barrel cortex to date. The other flatmap was used to showcase applications in data visualization.

Importantly, we have released an implementation of all our methods as free software, and made openly available the enhanced atlases and flatmaps obtained in our results (see Data and Code Availability).

While this is not the first time the concept of a flatmap has been introduced, we have described a general method that applies to any brain region with a principal axis of organization. Unlike flatmaps of a more schematic nature (Hahn et al., 2021), the type of data-driven flatmap produced by our method is adapted to the geometry of the voxelized atlas it is derived from. This enables a direct representation of data registered to the atlas (seeSection 3.4), similar to the flatmaps inWang et al. (2020)andWu et al. (2022)for mouse isocortex. Our approach differs from theirs as we use an area-preserving algorithm to parameterize the flat space, instead of a distance-preserving method with two reference points in the former, and an anatomical axis-based mapping in the latter. Our methodology also diverges from that ofDiedrichsen and Zotow (2015), as our flat space comprises a continuous mesh, contrasting with their introduction of surface cuts to accommodate human cerebellar anatomy, leading to a discontinuous flatmap.

One important thing to consider is that any flatmap, no matter how it is generated, introduces some degree of distortion. It is thus important to characterize this distortion to have an understanding of the biases implied when using the flatmap. For our main use case of barrel column annotations, we chose an area-preserving flatmap to ensure the flat projection of the barrels would map back nicely to 3D shapes, and also because it showed continuity and uniformity in the region of interest (barrel cortex). For other use cases, different properties may be desirable in a flatmap, such as having a natural border for visualization, and these choices can be guided by flatmap metrics providing qualitative and quantitative evaluation. A comprehensive side-by-side comparison between four mouse isocortex flatmaps (our two plus two published ones) can be found inSupplementary Material C. The comparison showed similar results for all flatmaps in terms of 2D representation of 3D properties such as region sizes, region border sizes, and geometric alignment between neighboring regions.

Our method generates flatmaps that associate each voxel with a single flat location. This property ensures that the preimages of nonoverlapping areas in flat space do not overlap themselves, which enables volume decomposition. In contrast, path-based approaches (e.g.,Wang et al., 2020) map a voxel to more than one pixel, which results in increased uniformity of preimage sizes (seeSupplementary Fig. 2B), but precludes volume decomposition. Using our flatmap, we partitioned the rat somatosensory cortex into nonoverlapping hexagonal subvolumes of approximately equal size. These subvolumes can be used to group spatially distributed data, and perform statistical analyses that could reveal functional gradients along the horizontal axes. A similar approach is applied inGao et al. (2022), to the mouse prefrontal cortex (PFC), in order to analyze the modular structure of intra-PFC connectivity. Furthermore, in the context of data-driven modeling, subvolumes can be extracted to run simulations of smaller parts of a larger model (Isbister et al., 2023) or used to characterize connectivity across scales (Reimann et al., 2022).

Despite the numerous studies in rodent barrel cortex, previous efforts on barrel segmentation have been made only in rat (Egger et al., 2012), and existing mouse atlases lack annotations of individual barrels. We used one of our flatmaps to derive annotations of barrels and their associated barrel columns with some notable properties. Firstly, they match previous accounts of barrels (Lefort et al., 2009;Welker & Woolsey, 1974) in terms of shapes and sizes. Secondly, they are defined in the standardized CCFv3 space, which enables the integration and analysis of datasets with single-barrel specificity. Finally, the barrel columns are nonoverlapping and follow the curvature of the cortex, resulting in a more biologically accurate representation than would be given by intersecting straight cylinders. We attribute these properties to our choice of flattening algorithm and the smoothness of the input atlas.

Data visualization in flat space is similar to the staining of mechanically flattened tangential sections of cortex. This classic technique has been applied even in a per-layer fashion in previous work (Fig. 13 ofPaperna & Malach, 1991, compared withFig. 7C). However, unlike mechanical flattening, where “angular parts of the cortex undergo a certain distortion, which may lead to misinterpretation of the location of labeled cells” (Paperna & Malach, 1991), our approach avoids this curvature bias by taking into account the local orientation at each point. Another advantage of digital methods like ours over mechanical flattening is the ease of visualization of data from different individuals registered to the same reference space.

Topographical mapping in flat space (Fig. 7A) has also been previously reported. For example, a flatmap of axonal projections between entorhinal cortex and dentate gyrus of rat (Dolorfo & Amaral, 1998) was used to define connections in a computational model of hippocampus (Hendrickson et al., 2016). Flatmaps are also a key component of an algorithm used to predict a long-range microconnectome of the whole mouse neocortex (Reimann et al., 2019).

Notwithstanding the successes of our method, it has some limitations. Notably, it is sensitive to edge artifacts. For example, in both atlases used in our results, some of the voxels at the edges of the source volume were not eligible. Since their numbers were very small compared with the total number of voxels, we did not try to fix them, but nevertheless these are locations where our method failed to assign three-dimensional coordinates. These edge artifacts are usually a consequence of discontinuities in the relative depth or orientation fields, or of noise present in the atlas, and thus can in general be diminished by using a smoother atlas. Even when that is not a possibility, one strategy to mitigate edge artifacts could be to consider a larger source volume, where possible, to effectively displace the edges away from the regions of interest. Alternatively, one could adopt a weaker eligibility criterion and keep all voxels whose streamlines reach the projection surface, even if these do not span the full depth. Ultimately, the missing coordinates could be filled in by extrapolation from nearby voxels.

Moreover, based on our flatmap metrics (Figs. 4Fand5E), locations with the largest nonuniformity and highest probability of noncontinuity were also at the periphery of flat space. We considered whether the stretching by the flattening algorithm of the projection mesh onto a convex polygon, that is, its boundary being made into straight lines regardless of its original shape, was to blame. We found this not to be the case, as we observed similar features in the periphery of both our flatmaps and in published flatmaps with curved borders (seeSupplementary Figs. 2B and 6A), so the main factor remains the above-mentioned impact of edge artifacts in the atlas. It is true, however, that artifacts may arise from the border definition process if the border has too many line segments compared with the number of vertices on the mesh border. It is thus recommended to either perform a simplification of the convex polygon or, if a detailed shape is required, to approximate it instead with a convex parametric curve to which the mesh border can be mapped smoothly.

One key parameter that can impact the quality of the flatmap is the depth$d^{*}$at which the projection mesh is defined. Given how the flatmapping algorithm works, area preservation and continuity of the flat space are best achieved at the selected depth, but as one moves away from$d^{*},$these properties can degrade. This means the flatmap has higher fidelity of representation for voxels close to the projection mesh, and lower the farther away from it. In our results, we placed the projection mesh in the exact middle of the source volume (approximately at layer 4), but use cases that focus on more superficial or deeper layers could benefit from a different choice of$d^{*}$(seeSupplementary Fig. 1A).

Another limitation stems from the use of streamlines to project voxels to the projection mesh. On the one hand, this ties the quality of the voxel projections to the quality of the orientation field, which may suffer from edge artifacts and discretization effects from limited resolution. On the other hand, while it is desirable that streamlines follow closely the geometry of a region, they are unavoidably subject to deformation depending on the relative size of the top and bottom shells at each location. For example, in slices of mouse prefrontal cortex, the top of layer 1 can be seen to be more than four times wider than the bottom of layer 6 (Van De Werd et al., 2010); this geometrical narrowing underlies the “hotspots” visible in our flatmap metrics of mouse isocortex (Fig. 3.2E) as well as the difference in size of prefrontal areas (especially the frontal pole) in our flatmaps compared with the AIBS flatmap.

Beyond its limitations, our method offers flexibility in both implementation and applicability to diverse use cases. We have already shown that our flatmapping algorithm can be applied to data produced by other groups using other methods (Wang et al., 2020; seeSection 3.2), as long as auxiliary atlases of local relative depth and orientation are available. If they are not, different approaches from ours can also be employed.

An alternative approach to generate relative depth and orientation fields is to solve the 3D heat equation inside the source volume, with “hot” top and “cold” bottom shells, and adiabatic sides (Airey et al., 2005;Jones et al., 2000;Lerch et al., 2008); then, the solution itself can be used as the relative depth field and its gradient as the local orientation (seeSection 3.2). Another similar, but simpler, approach consists in assigning the values 0 and 1 to the top and bottom shells, respectively, and then applying 3D Gaussian blur iteratively to distribute these values smoothly throughout the source volume. This last approach can also be used to generate a smooth field when layer boundaries do not coincide with level surfaces of the relative depth field, by assigning intermediate values in(0,1)to each layer boundary and smoothing in between them.

All of our as well as these additional approaches (includingWang et al., 2020) require marking the top and bottom shells of the source volume. While this can be achieved by manual selection (as shown), it can become an error-prone and time-consuming task depending on the complexity of the geometry (think hippocampus), and (semi-)automated approaches can be potentially very useful. One approach could be to use surface segmentation methods to partition the boundary mesh of the source volume into top, bottom, and side surfaces (Nuvoli et al., 2021;Shamir, 2008), and use these to guide voxel selection.

Regarding the flatmapping algorithm, while we have used specific algorithms in each step (and implementations thereof, seeSupplementary Material E), other algorithms can be used that achieve the same purpose. For example, there are many approaches in the field of computational geometry for level set extraction, surface reconstruction, mesh refinement and mesh flattening. The choice of alternatives by the user can serve to accommodate particular use cases or data. Also, while we have produced flatmaps that span large regions of cortex, for use cases that focus on single or small sets of cortical areas, it is possible to extract smaller parts of the projection mesh and use them to generate flatmaps restricted to those areas.

Applications of flatmaps usually work at a certain pixel resolution, and our method allows the choice of the best one for each use case, using our metrics as guidelines. For visualization purposes, the primary concern is to avoid the presence of empty preimages, that is, holes in the flat view. Other use cases, such as volume decomposition and definition of barrel columns, work with shapes drawn on flat space and can be conducted at native resolution. Finally, use cases such as long-range axon visualization require spatial continuity of the flat view, which may be attained at a lower resolution (seeSupplementary Fig. 1B). This is especially crucial when gradients are considered in flat space (Guyonnet-Hencke & Reimann, 2023), as continuity guarantees they correspond to gradients in Cartesian space.

It is worth mentioning that it is also possible to use our method to generate flatmaps of brain regions having no laminar architecture, such as subcortical structures, as long as a principal axis can be meaningfully defined (seeSupplementary Material Dfor an example). A useful guideline to define the orientation of the principal axis is to consider the directions in which axons exit or enter the structure, as can be determined from 3D reconstructions of complete axons (e.g.,Economo et al., 2016).

We think laminar coordinate systems offer new opportunities for data-driven research. In the case of modeling, having a coordinate system adapted to the brain region being modeled enables capturing spatial features of biological complexity, such as the distribution of cell types or gradients of cell properties throughout the region. For example, the relative depth field can be used to define annotations of individual layers and to derive local distances to layer boundaries; together with the orientation field, this information can be used to place reconstructed neuronal morphologies accurately in a brain region, in terms of both location and orientation, leading to realistic anatomical models (e.g.,Reimann et al., 2022).

Furthermore, our new flatmap-derived annotations of individual barrels and barrel columns provide a reference framework for future studies in barrel cortex, such as anatomical quantification and connectivity tracing (corticocortical and thalamocortical), as well as data-driven modeling.

In conclusion, we strongly believe that open and user-customizable methods to generate laminar coordinate systems and flatmaps are an important asset for the neuroscience community. Our contribution in this direction provides a basis for additional improvements allowing future neuroscience studies to benefit from these tools and approaches.

## Supplementary Material

Supplementary Material

## Data Availability

Enhanced atlases and flatmaps of rodent cortex that we have produced are available in a public repository under the CC BY license:https://doi.org/10.5281/zenodo.8165004. Our code implementing the flatmapping method and the definition of barrel cortex annotations is available in a public repository under the Apache-2.0 license:https://github.com/BlueBrain/atlas-enhancement. Data from the Allen Mouse Brain Common Coordinate Framework (CCFv3) reused in this work can be found underhttps://download.alleninstitute.org/informatics-archive/current-release/mouse_ccf/: annotation/ccf_2022/annotation_10.nrrd average_template/average_template_10.nrrd cortical_coordinates/ccf_2017/isocortex_mask_10.nrrd cortical_coordinates/ccf_2017/laplacian_10.nrrd cortical_coordinates/ccf_2017/dorsal_flatmap_paths_10.h5
