# Supplementary material for: Enhancement of brain atlases with laminar coordinate systems: Flatmaps and barrel column annotations
Source: Imaging Neurosci (Camb). 2024 Jun 28;2:imag-2-00209. doi: 10.1162/imag_a_00209 (PMC12272257; doi:10.1162/imag_a_00209)
Supplement: Supplementary Material [file imag_a_00209-supp.pdf]

Supplementary Material

A. Atlas of rat somatosensory cortex

The atlas was built in-house as the first step of an atlas-based model building effort that culminated in a large-scale biophysically detailed model of somatosensory cortex of juvenile rat (Reimann et al., 2022).

The starting point was the digital files distributed with Paxinos & Watson's printed atlas of the adult rat brain (Paxinos & Watson, 2007), consisting of vector drawings of coronal slices with annotated region outlines. These individual slices were aligned, rasterized and interpolated, then combined to create an isotropic voxelized atlas (at 40 μm resolution) with region labels corresponding to the region and acronym index in the book. The somatosensory regions in one hemisphere were then extracted and hierarchically smoothed to create an atlas of rat somatosensory cortex. Finally, this atlas was downscaled by a factor of $2082/2150 = 0.96837$, based on the cortical thickness of the S1HL region as measured in slices, to approximate the size of a juvenile (P14) rat brain.

The final atlas consists of an array with $409 \times 608 \times 286$ voxels of size 38.7348 μm. In total there are 10 annotated regions, including primary somatosensory representations of barrel field (S1BF), front limb (S1FL), hind limb (S1HL), jaw (S1J), shoulder (S1Sh), trunk (S1Tr), upper lip (S1ULp), dysgranular zone (S1DZ) and dysgranular oral zone (S1DZO); as well as secondary somatosensory region (S2) and an unspecified primary somatosensory region (S1).

B. Impact of some flatmapping parameters

We illustrate the impact of some parameters of the flatmapping algorithm. Namely, the choice of d^* and the interplay between native resolution (mesh refinement) and pixel resolution (discretization).

C. Comparison of mouse isocortex flatmaps

We performed a four-way side-by-side comparison between our two flatmaps of mouse isocortex (labeled as "Square" and "Shape-match"), the CCFv3 flatmap (Wang et al., 2020, labeled as "AIBS") and another published flatmap derived from the CCFv3 (Wu et al., 2022, labeled as "Kim Lab"), all at 10 μm resolution. The AIBS flatmap is path-based, meaning every flat position has associated a path that touches several voxels, whereas all others are voxel-based, meaning each voxel is mapped directly to a flat position. The "Kim Lab" flatmap was extracted from openly available data and put into the same

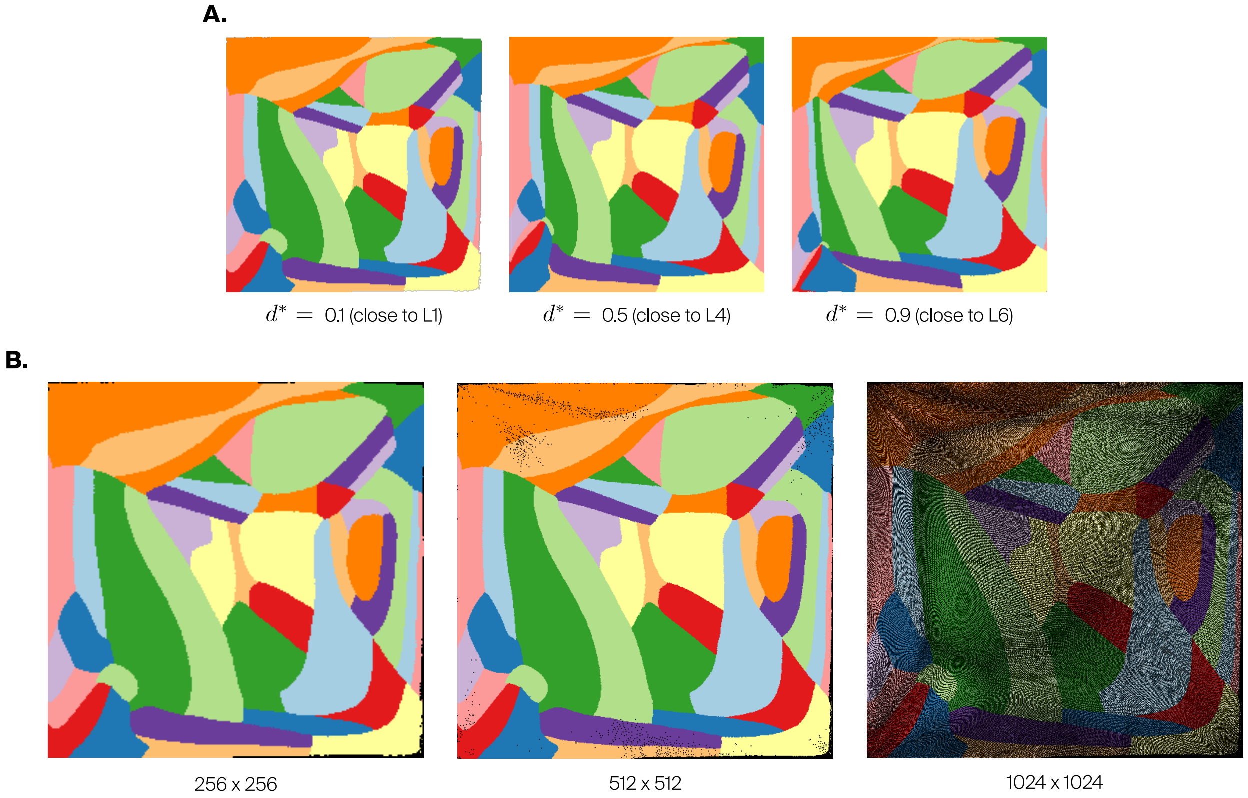

Supplementary Figure 1: **Impact of some parameters of the flatmapping algorithm. A.** Choice of d^* affects region sizes in flat space. From left to right: $d^* = 0.1$ (close to L1), 0.5 (close to L4), 0.9 (close to L6). Notice in particular how the frontal pole changes in size due to the divergence of streamlines. Region locations change slightly due to misalignment of flat axes between the three flatmaps. **B.** Interplay between mesh refinement and pixel resolution. Unrefined mesh exhibits holes (black background) at higher pixel resolutions. From left to right: pixel resolution 256x256, 512x512 and 1024x1024.

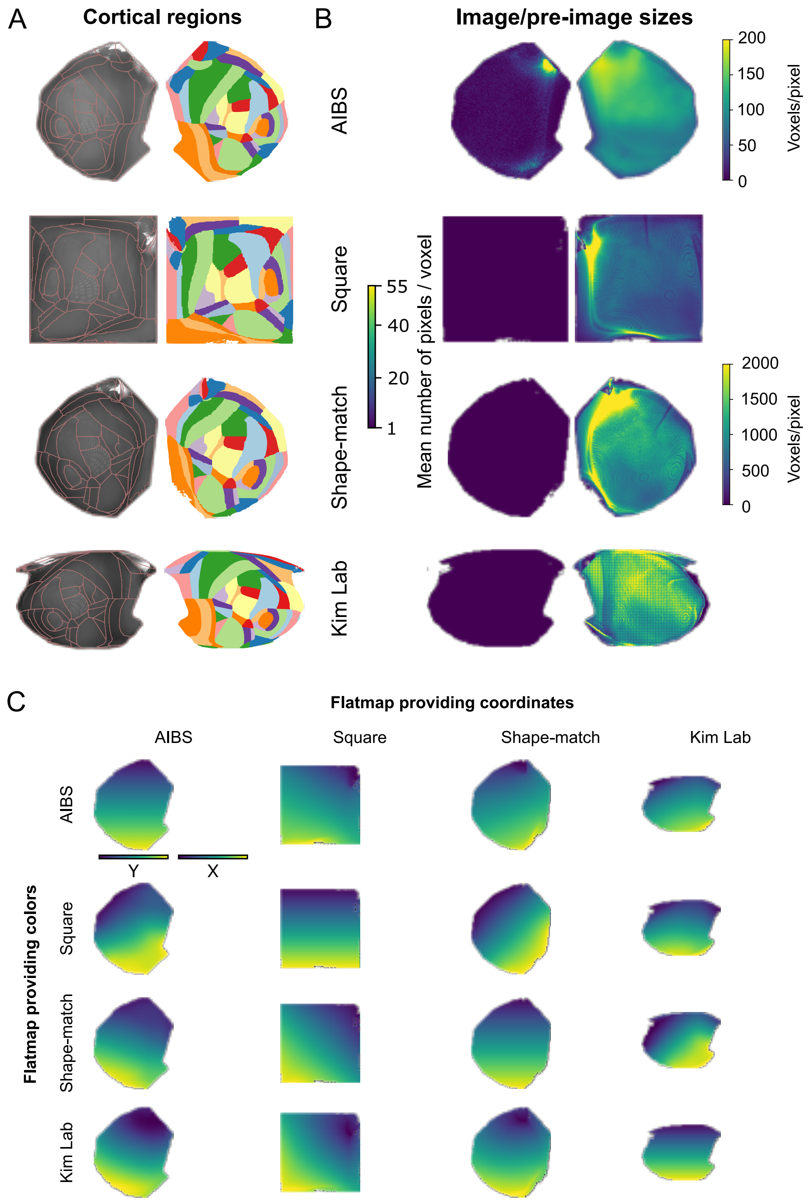

Supplementary Figure 2: **Qualitative flatmap comparison. A.** In each row we plot two data sources, displayed over one or the other hemisphere. Left: average template with region outlines; Right: flat view of region annotations (mode aggregation function). **B.** Same layout as in A. Left: heatmap of mean pixels per voxel; Right: heatmap of pre-image sizes (voxels per pixel). Only the "AIBS" flatmap maps a voxel to more than one pixel, specially in the frontal pole and medial regions, and has a constrained range of voxels per pixel by construction. Non-uniformities are evident in all flatmaps. **C.** Representation of flat coordinates of one flatmap in another flatmap. A column in the table gives the source flatmap and a row gives the destination flatmap. In each image, the Y coordinate is represented on the left hemisphere, and the X coordinate on the right hemisphere. Along the diagonal we observe the native coordinates of each flatmap, with Y from top to bottom and X from left to right, while off-diagonal images exhibit the differences in coordinate mappings across flatmaps.

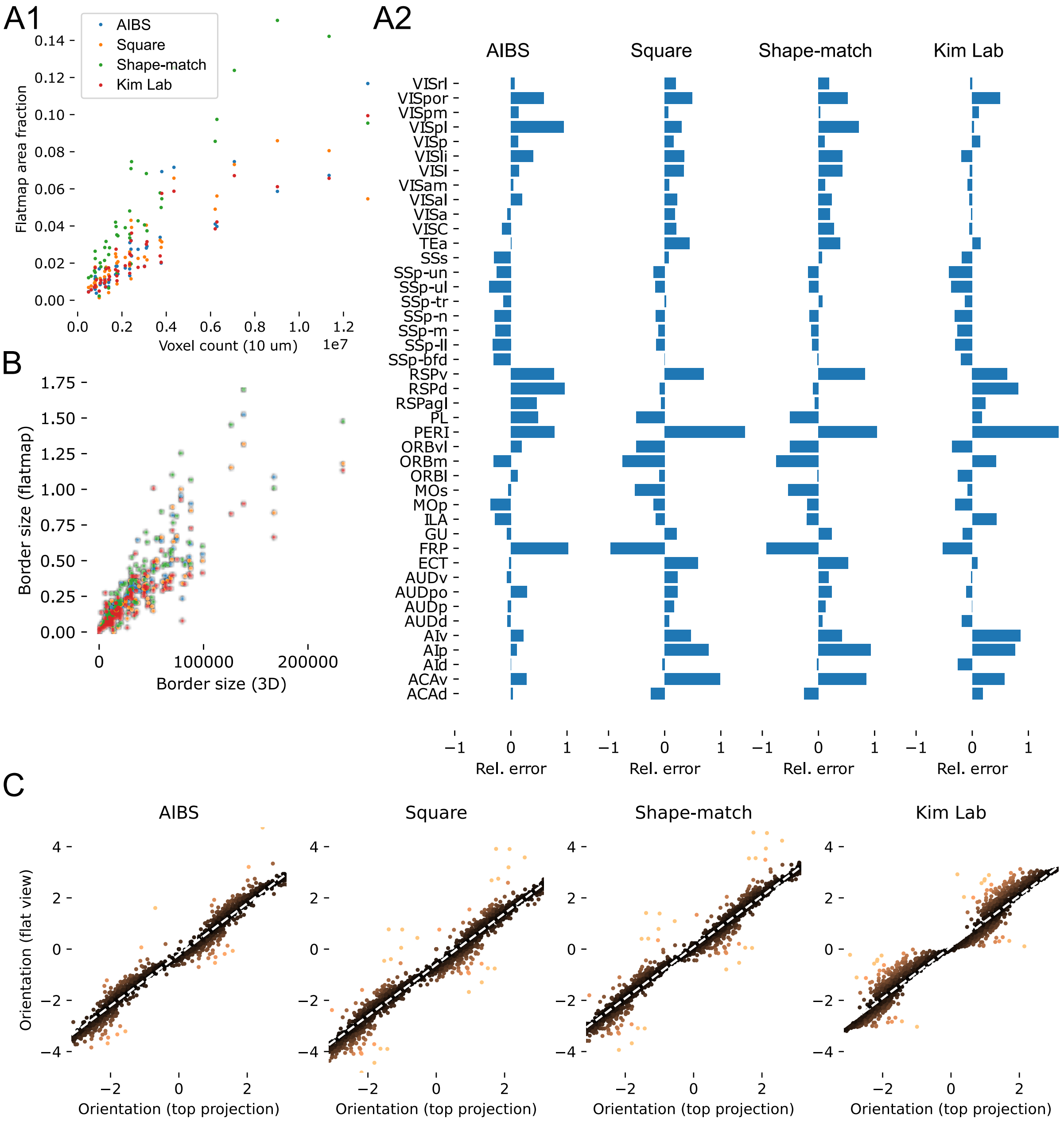

Supplementary Figure 3: **Quantitative flatmap comparison. A1.** Flat area (relative to whole flatmap) vs voxel count for each region. An approximate linear relation can be observed for in flatmaps. **A2.** Relative errors of flat areas vs voxel counts, computed based on the residuals of linear fits to A1 for each flatmap. We observe that all flatmaps have errors larger in some regions than others. Generally, errors are larger in the frontal pole and medial regions, consistent with previous observations. **B.** Flat border sizes vs 3D (Cartesian) border sizes for each region. An approximate linear relation can be observed in all flatmaps. **C.** Orientation between pairs of regions in flat space vs orientation in a top projection (a proxy for orientation in Cartesian space). We observe an approximate linear relation in all flatmaps.

format as our flatmaps to facilitate the comparison (MATLAB to NNRD conversion). For comparison purposes, we restricted our analyses to the common domain of voxels mapped by the "AIBS" flatmap (which samples sparsely the source volume with 68% coverage, at 10 μm resolution).

We devised some metrics to compare the quality of the geometrical representation of cortical regions across different flatmaps with the same source volume, in this case, the whole mouse isocortex in CCFv3 atlas. First we compare flat region annotations, image/pre-image size distributions and flat coordinates; then we analyze how well the flatmaps represent region areas, borders and orientations. A Python notebook is available to run these analyses (see Data and Code Availability).

We considered as the actual size of a brain region the number of voxels associated with it in the CCFv3 atlas (at 10 μm resolution). The flat size of a brain region was calculated as the number of pixels associated with it in a flat view of region annotations (e.g., Suppl. Fig. 3A1). For a given flat map, we computed a linear fit between the flat and actual sizes of all cortical regions, which provided for each flat size an implied actual size. We calculated for each region the difference between actual and implied sizes and normalized the results by their actual size, yielding a relative error (Suppl. Fig. 3A2).

We calculated the size of the border between neighboring regions (Suppl. Fig. 3B). Starting from a flat view of region annotations, for a given pixel associated with region i we considered its four direct neighbors and counted how many of them were associated with given region j. The sum of this value over all pixels associated with i is what we considered the length of the border between i and j. Note that this measure is symmetric. For the border in three dimensional space, we used the voxel annotations of CCFv3 and considered direct neighbors along the three axes (6 neighbors in total).

We evaluated the orientation of a pair of regions, i and j with respect to each other (Suppl. Fig. 3C). We found the centroid of both regions by averaging their X and Y coordinates in a flat view of region annotations. We then calculated the orientation of a line connecting the center of i to the center of j as $a = atan(\frac{\Delta X}{\Delta Y})$, where ΔX and ΔY were the difference between the X and Y coordinates of j and i, respectively. We compared the results over all pairs of regions between a given flatmap and the parallel projection from the top (i.e. by discarding the Y coordinate of the CCFv3). Then, we considered how well the two orientations matched after subtracting a global rotation. We optimized the angle of the global rotation as follows: Let $a_{i,j}^{fm}$ be the orientation for regions i and j in the flatmap, $a_{i,j}^{hor}$ their orientation in the horizontal plane. Then: $a_{opt} = \sum_{i,j} \frac{a_{i,j}^{fm} - a_{i,j}^{hor}}{N}$ minimizes the sum of absolute error when $a_{i,j}^{hor}$ is compared to $a_{i,j}^{fm} - a_{opt}$. Finally, we evaluated the remaining mean absolute error over all pairs of regions for a given flatmap.

D. Flatmapping of non-layered brain structures

Our flatmapping method can also be applied to non-layered brain regions, as long as a principal axis can be defined that is meaningful for the geometry of the structure under study.

Here we provide as an example the generation of a flatmap for the dorsal striatum of mouse. This structure has no cytoarchitectonically defined layers, but it has a smooth organic shape with a meniscus cross-section (similar to a cupped hand) that can be approximated by an ellipsoid. The principal axis is then defined as the normal vector to the surface of the ellipsoid, and the flatmap axes go along the surface of the ellipsoid.

In detail:

1. We extract the voxels belonging to the dorsal striatum (STRd) region from the Allen Mouse Brain Common Coordinate Framework version 3 at 25 um resolution.

2. We fit a plane to the point cloud defined by the voxel centers, in order to get an idea of the "facing" direction of the structure.

3. We fit a sphere to the point cloud on the concave side, with center along the line defined by the plane normal.

4. Using the position and radius of the sphere as initial condition, we fit an ellipsoid using the sum of orthogonal distances as the cost function with the COBYLA optimization algorithm (NLopt library). The resulting ellipsoid passes through the "middle" of the point cloud, similar to the choice of isosurface with relative depth 0.5 in the main text.

5. We compute the bounding box of the point cloud and intersect this with the ellipsoid to obtain a patch of ellipsoidal surface that we use as projection mesh.

6. We compute the projections of voxel centers as orthogonal projection to the ellipsoid, that is, streamlines are assumed to be straight line segments having the orientation of the surface normals.

7. We compute relative depth by considering the normalized distance to the ellipsoid surface, taking into account which side of the surface the point is in, so we obtain a continuous value in [0,1].

8. The rest of the algorithm proceeds as usual, with authalic flattening of the projection mesh and finding nearest neighbors between intersection points and mesh vertices.

The resulting flatmap provides coordinates along the "face" of the dorsal striatum, and allows a decomposition of its volume into uniform subvolumes. A similar approach can be applied to any brain structure with a similar shape, e.g. reticular nucleus of thalamus, etc.

E. Implementation details

General

- All code was run under GNU/Linux 64-bit on the BB5 supercomputer hosted at the Swiss National Supercomputing Centre (CSCS) in Lugano.
- The workflow orchestrating all steps of the algorithm is written as a GNU Makefile.
- Handling of atlas volumes in NRRD format is performed in Python using `voxcell` (Povolotsky et al., 2023).

Flatmapping stage I: Flat mesh generation

- Extraction of the point cloud of voxels approximately at relative depth d^* is implemented in Python using the Euclidean Distance Transform (EDT) algorithm in SciPy.
- Reconstruction of the projection mesh is implemented in C++ using the Scale-Space Surface Reconstruction package from the Computational Geometry Algorithms Library (CGAL, van Lankveld, 2023).
- Refinement of the projection mesh by uniform subdivision is performed with GMSH (Geuzaine & Remacle, 2009).
- Flattening of the projection mesh is performed using the (Iterative) Authalic Mapping algorithm from the Triangulated Surface Mesh Parameterization CGAL package (Saboret et al., 2023).

Flatmapping stage II: Voxel projection

- Integration of streamlines is implemented in C using the numerical ODE integrator with explicit embedded Runge-Kutta (2,3) method from the GNU Scientific Library (GSL, Galassi, 2009).
- Parametrization of streamlines is implemented in C using linear spline interpolation from GSL.

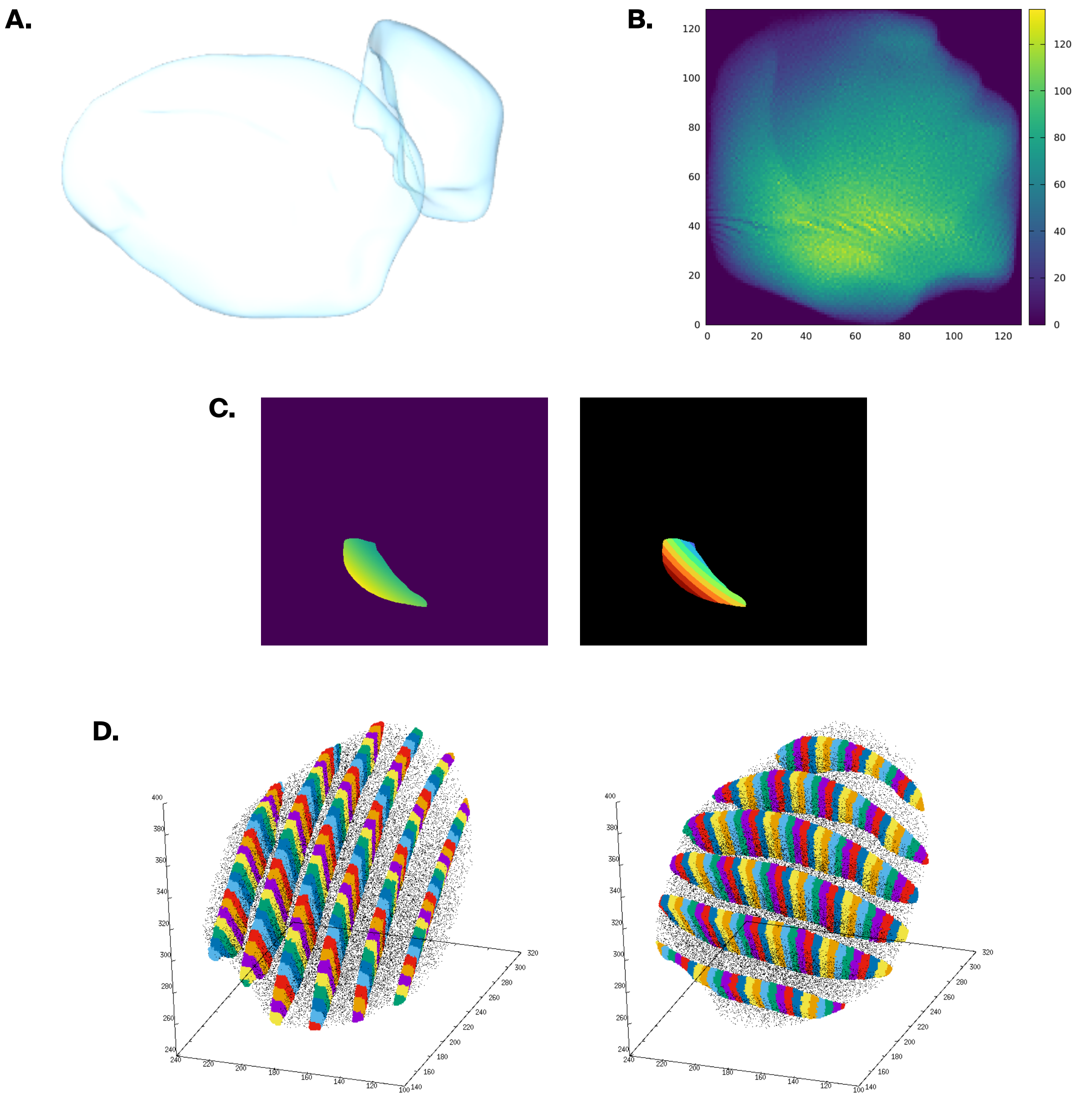

Supplementary Figure 4: **Flatmapping of non-layered brain structures: dorsal striatum. A.** 3D meshes of bilateral dorsal striatum, showing "face" of structure and lateral aspect. **B.** Pre-image size heatmap. Note how the edges of the flatmap do not coincide with the edges of the square due to how the projection mesh was chosen. **C.** Slice through the relative depth field, showing continuous variation (left) and discrete levels to exhibit ellipsoidal shape of isosurfaces (right). **D.** Decomposition of the volume of dorsal striatium into square columns. Note how the columns curve following the normals of the ellipsoidal surface and span the full depth.

- Projection of voxel centers to the projection surface is implemented in C using the Brent-Dekker one-dimensional root finding algorithm from GSL.
- Distributed parallel computation of voxel projections is performed with the help of GNU Parallel (Tange, 2011).

Flatmapping stage III: Flatmap generation

- Mapping of streamline intersections to mesh points is implemented in C++ using Nearest Neighbor search from the dD Spatial Search CGAL package (Tangelder & Fabri, 2023).
- Discretization of the flatmap is performed in Python using NumPy.

Applications

- Plotting of flat views is performed in Python using Datashader.

F. Radial volume distribution of a truncated square pyramid

When computing the width of the pre-image of a pixel, we perform a fit based on the radial volume distribution of the pre-image, using the best-fit line through the centroid as axis. We fit the two radii of a truncated square pyramid based on the analytical expression for the normalized radial volume distribution, which we describe here.

Essentially, we are computing the volume of the intersection between a truncated square pyramid with radii r_0, r_1 ($r_0 \leq r_1$) and height H, and a cylinder of radius r with the same axis. To compute this volume as a function of r, we take the integral along height of the cross-sectional area of the intersection.

The cross-section at any point corresponds to the intersection between a circle of radius r and a square of radius a concentric to it, which has an area (Suppl. Fig. 5A):

$$A(r;a) = \begin{cases} \pi r^2 & r \leqslant a \\ \pi r^2 - 4\left(r^2\, arctan\left(\frac{\sqrt{r^2-a^2}}{a}\right) - a\sqrt{r^2-a^2}\right) & a < r \leqslant \sqrt{2}a \\ 4a^2 & r > \sqrt{2}a \end{cases} . \tag{1}$$

In the case of the truncated square pyramid, a depends linearly on height:

$$a(h) = r_0 + Kh$$

with $K = \frac{r_1 - r_0}{H}$, so we can write the volume integral as:

$$\begin{aligned} V(r) &= \int_0^H A(r; a(h))dh \\ &= \frac{1}{K}\int_{r_0}^{r_1} A(r; u)du. \end{aligned}$$

Now, since $A(r; u)$ is defined piecewise, this integral ends up split into intervals based on the range of r relative to u. In any case, the result is a combination of the definite integrals of the three cases in (1), which are given by (Suppl. Fig. 5B):

$$V_c(r; a, b) = \frac{b-a}{K}\pi r^2,$$

the volume of a cylinder of radius r and height $H = \frac{b-a}{K}$,

$$\begin{aligned} V_q(r; a, b) &= V_p(r; b) - V_p(r; a) \\ V_p(r; u) &= \frac{\pi r^2}{K}u + \frac{4}{3K}\left[\sqrt{r^2 - u^2}(u^2 + 2r^2) - 3r^2\, arctan\left(\frac{\sqrt{r^2-u^2}}{u}\right)u\right], \end{aligned}$$

the volume of a proper intersection between a cylinder of radius r and a truncated square pyramid with radii a, b ($b \geq a$), and

$$V_f(a, b) = \frac{4}{3K}(b^3 - a^3),$$

the full volume of a truncated square pyramid with radii a, b ($b \geq a$) and height $H = \frac{b-a}{K}$.

The following special cases of $V_p(r; u)$ are useful for numerical evaluation:

$$V_p(r; r) = \frac{\pi}{K}r^3 \qquad\qquad V_p\left(r; \frac{r}{\sqrt{2}}\right) = \frac{5\sqrt{2}}{3K}r^3.$$

In terms of these integrals, and considering the different ranges of r, we write the radial volume distribution of a truncated square pyramid with radii r_0, r_1 $(r_0 \leq r_1)$ and height $H = \frac{r_1 - r_0}{K}$ as:

$$V(r) = \begin{cases} V_c(r; r_0, r_1) & r \leqslant r_0 \\ V_q(r; r_0, r) + V_c(r; r, r_1) & r_0 < r \leqslant \min\left(\sqrt{2}r_0, r_1\right) \\ V_f(r_0, \frac{r}{\sqrt{2}}) + V_q(r; \frac{r}{\sqrt{2}}, r) + V_c\left(r; r, r_1\right) & \sqrt{2}r_0 < r \leqslant r_1 \\ V_q(r; r_0, r_1) & r_1 < r \leqslant \sqrt{2}r_0 \\ V_f(r_0, \frac{r}{\sqrt{2}}) + V_q(r; \frac{r}{\sqrt{2}}, r_1) & \max\left(\sqrt{2}r_0, r_1\right) < r \leqslant \sqrt{2}r_1 \\ V_f(r_0, r_1) & \sqrt{2}r_1 < r \end{cases} .$$

The third and fourth cases are exclusive to one another, with the third one occurring only when $r_1 > \sqrt{2}r_0$ and the fourth one in the opposite case. When $r_1 \to r_0$, the function tends to the volume of the square prism.

By considering the quotient with the total volume, i.e. $V(r)/V_f(r_0, r_1)$, we obtain the volume fraction, a function with values in $[0, 1]$ that only depends on r_0 and r_1, but not on H (since all terms have a factor of $\frac{1}{K}$ that cancels out when taking the quotient). This function can be readily implemented numerically and fitted using a least squares approach to the empirical cumulative distribution function (CDF) of the data (Suppl. Fig. 5C).

G. Flatmap metrics for shape-match flatmap

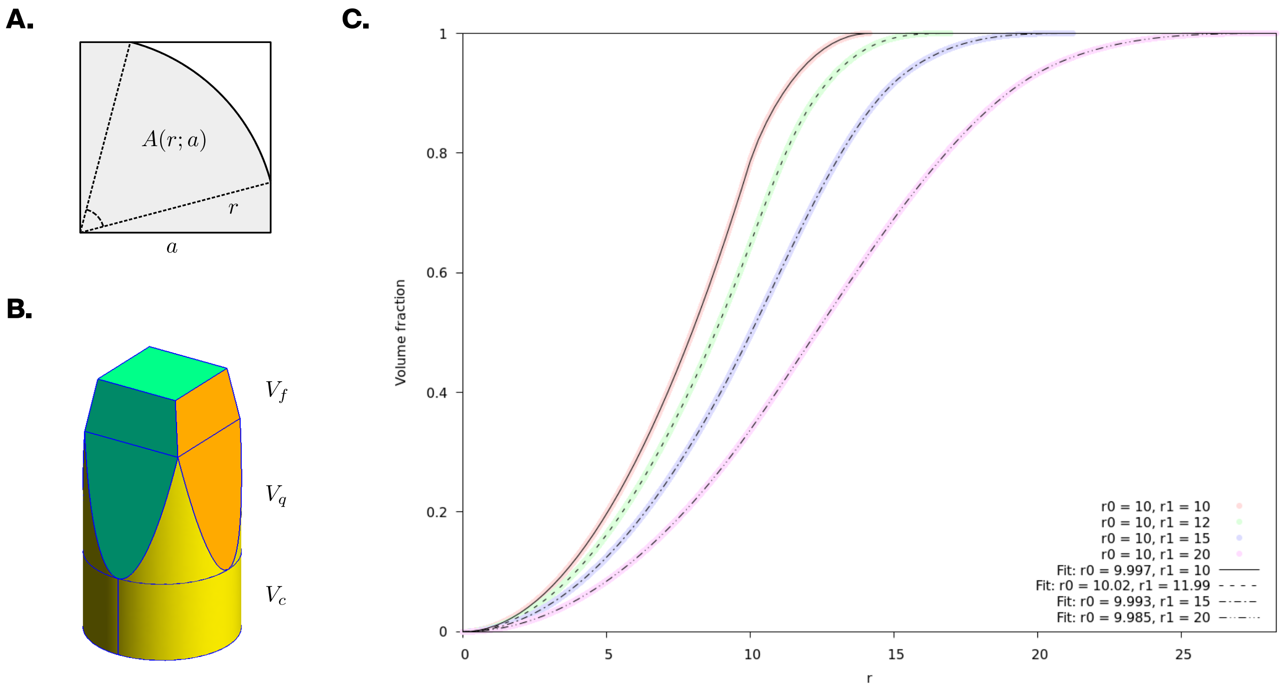

Supplementary Figure 5: **Radial volume distribution of a truncated square pyramid. A.** Geometry of the intersection between a circle of radius r and a square of radius a, from which $A(r;a)$ in Equation (1) can be derived using basic trigonometry. **B.** 3D schematic of the volumes described by the three cases of the volume integral: truncated square pyramid (V_f, top), cylinder (V_c, bottom), and their intersection (V_q, middle). **C.** Least squares fits of the normalized radial volume distribution to the empirical CDF of data generated by random sampling of a perfect truncated square pyramid with given radii. An excellent fit is obtained in all cases: $r_1 = r_0$, $r_1 < \sqrt{2}r_0$ and $r_1 > \sqrt{2}r_0$, providing empirical evidence of the applicability of our analytical derivation.

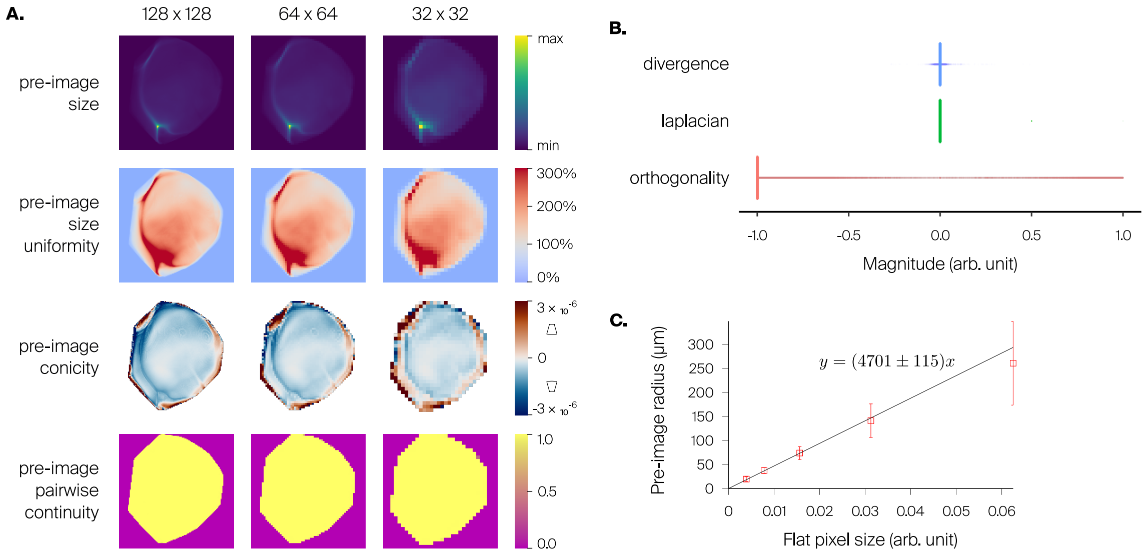

Supplementary Figure 6: **Flatmap metrics for shape-match flatmap.** **A.** Per-pixel metrics. **B.** Per-voxel metrics. **C.** Distance metric.

References

Galassi, M. (Ed.). (2009). *GNU scientific library reference manual: For GSL version 1.12* (3rd). Network Theory.

Geuzaine, C., & Remacle, J.-F. (2009). Gmsh: A 3-D finite element mesh generator with built-in pre- and post-processing facilities. *International Journal for Numerical Methods in Engineering*, *79*(11), 1309–1331. https://doi.org/10.1002/nme.2579

Paxinos, G., & Watson, C. (2007). *The rat brain in stereotaxic coordinates* (6th). Elsevier.

Povolotsky, A. V., MikeG, Luis, asanin-epfl, jdcourcol, Guyot, L., Ficarelli, G., Zisis, E., Vanherpe, L., HDictus, alex4200, Berchet, A., O'Reilly, C., Šubert, E., & Antonel, S. (2023, November). *Blue-brain/voxcell: V3.1.6* (Version v3.1.6). Zenodo. https://doi.org/10.5281/zenodo.10171413

Reimann, M. W., Bolaños-Puchet, S., Courcol, J.-D., Santander, D. E., Arnaudon, A., Coste, B., Delemontex, T., Devresse, A., Dictus, H., Dietz, A., Ecker, A., Favreau, C., Ficarelli, G., Gevaert, M., Hernando, J. B., Herttuainen, J., Isbister, J. B., Kanari, L., Keller, D., ... Markram, H. (2022). Modeling and Simulation of Rat Non-Barrel Somatosensory Cortex. Part I: Modeling Anatomy. *bioRxiv*. https://doi.org/10.1101/2022.08.11.503144

Saboret, L., Alliez, P., Lévy, B., Rouxel-Labbé, M., Fabri, A., & Jain, H. (2023). Triangulated surface mesh parameterization. In *CGAL user and reference manual*. CGAL Editorial Board. https://doc.cgal.org/5.5.2/Manual/packages.html#PkgSurfaceMeshParameterization

Tange, O. (2011). GNU Parallel - The Command-Line Power Tool. *;login: The USENIX Magazine*, 42–47.

Tangelder, H., & Fabri, A. (2023). dD spatial searching. In *CGAL user and reference manual*. CGAL Editorial Board. https://doc.cgal.org/5.5.2/Manual/packages.html#PkgSpatialSearchingD

van Lankveld, T. (2023). Scale-space surface reconstruction. In *CGAL user and reference manual*. CGAL Editorial Board. https://doc.cgal.org/5.5.2/Manual/packages.html#PkgScaleSpaceReconstruction3

Wang, Q., Ding, S.-L., Li, Y., Royall, J., Feng, D., Lesnar, P., Graddis, N., Naeemi, M., Facer, B., Ho, A., Dolbeare, T., Blanchard, B., Dee, N., Wakeman, W., Hirokawa, K. E., Szafer, A., Sunkin, S. M., Oh, S. W., Bernard, A., ... Ng, L. (2020). The Allen Mouse Brain Common Coordinate Framework: A 3D Reference Atlas. *Cell*, *181*(4), 936–953.e20. https://doi.org/10.1016/j.cell.2020.04.007

Wu, Y.-t., Bennett, H. C., Chon, U., Vanselow, D. J., Zhang, Q., Muñoz-Castañeda, R., Cheng, K. C., Osten, P., Drew, P. J., & Kim, Y. (2022). Quantitative relationship between cerebrovascular network and neuronal cell types in mice. *Cell Reports*, *39*(12), 110978. https://doi.org/10.1016/j.celrep.2022.110978
